# Unagi: Deep Generative Model for Deciphering Cellular Dynamics and In-Silico Drug Discovery in Complex Diseases

**DOI:** 10.21203/rs.3.rs-3676579/v1

**Published:** 2023-12-18

**Authors:** Yumin Zheng, Jonas C. Schupp, Taylor Adams, Geremy Clair, Aurelien Justet, Farida Ahangari, Xiting Yan, Paul Hansen, Marianne Carlon, Emanuela Cortesi, Marie Vermant, Robin Vos, Laurens J. De Sadeleer, Ivan O Rosas, Ricardo Pineda, John Sembrat, Melanie Königshoff, John E. McDonough, Bart M. Vanaudenaerde, Wim A. Wuyts, Naftali Kaminski, Jun Ding

**Affiliations:** 1 Quantitative Life Sciences, Faculty of Medicine & Health Sciences, McGill University, Montreal, QC, Canada.; 2 Meakins-Christie Laboratories, Translational Research in Respiratory Diseases Program, Research Institute of the McGill University Health Centre, Montreal, QC, Canada; 3 Pulmonary, Critical Care and Sleep Medicine, Yale University, School of Medicine, New Haven, CT, United States.; 4 Biological Sciences Division, Pacific Northwest National Laboratory, Richland, WA, United States.; 5 Laboratory of Respiratory Diseases and Thoracic Surgery (BREATHE), Department of Chronic Diseases and Metabolism, KU Leuven, Belgium; 6 Division of Pulmonary, Critical Care and Sleep Medicine, Baylor College of Medicine, Houston, TX, USA; 7 Division of Pulmonary, Allergy, Critical Care and Sleep Medicine, Department of Medicine, University of Pittsburgh, Pittsburgh, PA, USA; 8 Mila - Quebec AI Institute, Montreal, QC, Canada

## Abstract

Human diseases are characterized by intricate cellular dynamics. Single-cell sequencing provides critical insights, yet a persistent gap remains in computational tools for detailed disease progression analysis and targeted in-silico drug interventions. Here, we introduce UNAGI, a deep generative neural network tailored to analyze time-series single-cell transcriptomic data. This tool captures the complex cellular dynamics underlying disease progression, enhancing drug perturbation modeling and discovery. When applied to a dataset from patients with Idiopathic Pulmonary Fibrosis (IPF), UNAGI learns disease-informed cell embeddings that sharpen our understanding of disease progression, leading to the identification of potential therapeutic drug candidates. Validation via proteomics reveals the accuracy of UNAGI’s cellular dynamics analyses, and the use of the Fibrotic Cocktail treated human Precision-cut Lung Slices confirms UNAGI’s predictions that Nifedipine, an antihypertensive drug, may have antifibrotic effects on human tissues. UNAGI’s versatility extends to other diseases, including a COVID dataset, demonstrating adaptability and confirming its broader applicability in decoding complex cellular dynamics beyond IPF, amplifying its utility in the quest for therapeutic solutions across diverse pathological landscapes.

Complex diseases emerge through the interaction of genetic and environmental factors over time. The complexity of the interactions between these heterogeneous factors among individuals and populations challenges the understanding of disease progression^1–3^. Treating multifactorial diseases requires therapies that address multiple interacting processes, which complicates and prolongs the drug development process^4,5^. The lack of understanding of disease cellular dynamics poses challenges to the effectiveness of therapeutic targets and developed drugs, as most of them are developed in animal or cell culture models that ignore the complexity and dynamics of the human disease.

Single-cell RNA sequencing (scRNA-seq) stands at the frontier of potential solutions, offering an unprecedented opportunity to analyze cell populations at single-cell resolution^6,7^, and profile the complex and heterogeneous systems^8,9^, thereby uncovering rare cell populations and aberrant cell states that are pivotal to diseases^10^. Computation methods including Seurat^11^, Scanpy^12^, Monocle 3^13^, Cellar^14^ and scVI^15^ could analyze the noisy, high-dimensional, and large-scale scRNA-seq data and sketch cellular dynamics. However, scRNA-seq data is often a snapshot of the cellular states at a specific time point and cannot account for the continuous biological process, such as differentiation or immune responses, during the progression of a disease. Time-series scRNA-seq data can enhance our grasp on the regulatory mechanisms underpinning disease progression based on distinct samples from multiple time points^16^. Nevertheless, cell asynchrony presents new computational challenges to uncover the temporal cellular dynamics^8,16,17^. When applying snapshot-based scRNA-seq analysis tools to time-series data, they tend to perceive the data as discrete snapshots, overlooking the continuity and temporal progression inherent to time-series data. The omission of nuanced temporal information, like disease stage transitions, is not accurately modeled.

Computational methods have been developed to address the challenges raised by time-series single-cell transcriptome data, however both conventional methods such as scdiff^18^ and CSHMMs^19,20^, and deep learning-based methods such as RVAgene^21^ and TDL^22^ are engineered for generic single-cell data processing, inadvertently bypassing the specialized necessities tied to complex diseases. Rigorous preprocessing and normalization, often needed by noisy single-cell data for complex diseases, can shift the data into unconventional distributions, making them ill-suited for the direct application of many existing models on single-cell applications^15,23,24^. These techniques were often not originally designed to handle data that deviates from conventional distributions, leading to suboptimal or even inaccurate results. When it comes to the critical step of cell embedding learning, these methods typically employ a one-size-fits-all approach. Their dimensionality reductions and cell embedding strategies are largely generic, devoid of the flexibility to integrate disease-specific signatures or intricacies, rendering them less effective in capturing nuanced biological variances associated with complex diseases. Another salient gap in current single-cell methodologies is the absence of **unsupervised** *in-silico* exploration capabilities. Although GEARS^25^ and scGen^23^ are able to perform *in-silico* perturbations, they weren’t designed to process time-series data and require the *in-vitro* screening of perturbation response of disease and drug treatments as the supervision. Thus, there is a pressing need for methods that can virtually examine thousands of potential drugs and compounds on single-cell disease data without ground truth training data. The surge in large-scale public drug treatment databases, like the Connectivity Map (CMAP) database^26,27^, may provide the missing link to the unsupervised single-cell *in-silico* drug perturbations. Coupled with this, given the vast pool of drug candidates and the intricate cellular dynamics of diseases, an interactive visualization tool is indispensable for efficiently probing potential drugs and priming them for further experimental validation.

Addressing these pressing gaps, we present UNAGI, a comprehensive unsupervised *in-silico* cellular dynamics and drug discovery framework. UNAGI deciphers cellular dynamics from human disease time-series single-cell data and facilitates *in-silico* drug perturbations to earmark therapeutic targets and drugs potentially active against complex human diseases. All outputs, from cellular dynamics to drug perturbations, are rendered in an interactive visual format within the UNAGI framework. Nestled within a deep learning architecture Variational Autoencoder-Generative adversarial network (VAE-GAN), UNAGI is tailored to manage diverse data distributions frequently arising post-normalization. It also employs disease-informed cell embeddings, harnessing crucial gene markers derived from the disease dataset. On achieving cell embeddings, UNAGI fabricates a graph that chronologically links cell clusters across disease stages, subsequently deducing the gene regulatory network orchestrating these connections. UNAGI is primed to leverage time-series data, enabling a precise portrayal of cellular dynamics and capture of disease markers and gene regulators. Lastly, the deep generative prowess of the UNAGI framework powers an *in-silico* drug perturbation module, simulating drug impacts by manipulating the latent space informed by real drug perturbation data from the CMAP database. This allows for an empirical assessment of drug efficacy based on cellular shifts towards healthier states following drug treatment. The in-silico perturbation module can similarly be utilized to investigate therapeutic pathways, employing an approach akin to the one used in drug perturbation analysis.

We demonstrate UNAGI on a comprehensive single-nuclei RNA-seq (snRNA-seq) IPF dataset. IPF is a complex lethal lung disease characterized by irreversible lung scarring, leading to progressive decline in lung function and death^28–30^. Present therapeutic options for IPF are markedly narrow; only two FDA-approved medications, Pirfenidone^31^ and Nintedanib^32^, exist, and their main effect is slowing lung function decline, not reversing fibrosis, or making the patient feel better^33^. Despite their approval, their specific impact on disease mechanisms remains unclear^32–34^. Recent studies^10,35^ highlighted the molecular and cellular diversity of the IPF lung, but this information has not been yet incorporated in the development of therapies for IPF, although some studies suggested that computational analyses may identify potential drugs for human pulmonary fibrosis^36^. The push towards developing potent IPF therapeutics is hampered further by the incomplete understanding of the dynamic changes of diverse cellular populations throughout IPF progression. We apply UNAGI to a unique dataset that contains samples from differentially affected lung regions, allowing analysis of disease progression, and highlighting UNAGI’s ability to generate compact low-dimensional representations for subsequent tasks, outclassing existing methods. Further, we apply proteomics analysis and Precision-cut Lung Slices (PCLS) analysis^37,38^ to experimentally confirm the results and predictions of UNAGI. Taken together, our findings corroborate UNAGI’s capability not just in decoding cellular dynamics and underpinning regulatory networks but also in potentially accelerating drug development by spotlighting potential therapeutic targets and drug candidates.

## Results

### Overview of UNAGI conceptual framework

**Fig. 1 |:**
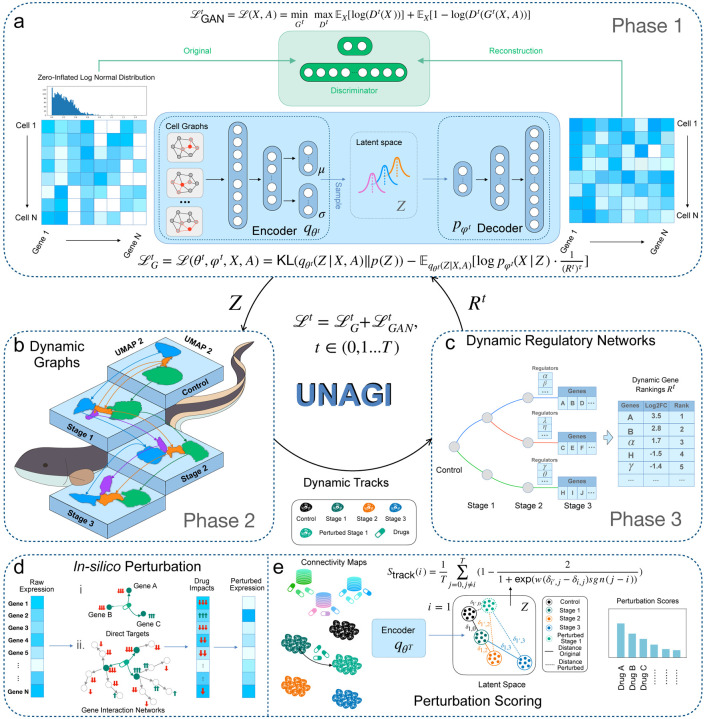
UNAGI overview: resolving cellular dynamics of complex disease & potential therapeutics through single-cell embeddings. **a,** Phase 1: UNAGI employs a VAE-GAN paired with a graph convolution layer. This setup harnesses the complexities of single-cell data, producing a ‘Z’ latent space that bridges encoding and decoding with minimal error. **b,** Phase 2: Derived from the ‘Z’ embeddings, a temporal dynamics graph emerges. Here, the Leiden clustering method discerns cell populations, subsequently connecting them across stages based on their inherent similarity. **c,** Phase 3: The iDREM tool comes into play, spotlighting key gene regulators and genes that influence disease progression. These insights are channeled into an iterative model training, honing in on specific gene markers of the disease. **d,** With the model in place, UNAGI initiates in-silico perturbations, either directly tweaking drug target gene expressions (i) or manipulating gene expressions via established gene interaction networks (ii) to simulate drug treatment impact. **e,** UNAGI’s encoder processes the perturbed cell population alongside its peers. The perturbation scores, derived from the ‘Z’ space embeddings generated by the UNAGI encoder, assist in identifying potential drug candidates. These candidates are evaluated based on their ability to transition diseased cells towards healthier states, such as those resembling healthy control cells, thereby contributing to the treatment of the disease.

UNAGI, **u**nified *i****n****-silico* cellular dyn**a**mics and dru**g** d**i**scovery framework, is a computational framework that integrates time-series single-cell sequencing data with sophisticated deep-learning techniques to unravel intricate cellular dynamics and identify potent therapeutic interventions against multifaceted diseases. This is achieved using the following three components: (1) UNAGI applies a VAE-GAN to capture cellular information in a reduced latent space (Fig. 1a). It processes single-cell data as continuous, zero-inflated log-normal (ZILN) distributions (or other distributions that well fit the data in other application scenarios) because this often better matches the distribution of single-cell data post rigorous preprocessing and normalization (e.g., in the IPF data employed in this study). With a cell-by-gene normalized counts matrix as input, a cell graph convolution (GCN) layer is introduced to manage the sparse and noisy nature of the data. Particularly, the GCN layer leverages the structured relationships between cells to mitigate the dropout noise common in single-cell data, enhancing the accuracy of cellular representations. This data, further refined by a VAE, results in lower-dimensional embeddings, with an adversarial discriminator ensuring the synthetic quality of these representations. (2) After embedding, cell populations are identified using the Leiden clustering approach and visualized with UMAP. A temporal dynamics graph is then constructed by evaluating cell population similarities during the disease progression, linking them based on their likeness (Fig. 1b). Each trajectory within the graph then forms the basis for deriving gene regulatory networks using the iDREM tool (Fig. 1c). (3) An iterative refinement process toggles between the embedding and temporal dynamics stages. Critical gene regulators, including transcription factors, cofactors, and epigenetic modulators, identified from the temporal cellular dynamics reconstruction stage are emphasized during the subsequent embedding phase, ensuring that the cell representation learning places heightened focus on these pivotal elements related to disease progression in each iteration. (4) Upon reaching predefined stopping criteria, UNAGI then employs *in-silico* perturbations to quantify the effectiveness of therapeutic interventions (Fig. 1d). Using the trained VAE-GAN generative model, UNAGI simulates cells under various drug treatments or pathway perturbations. Each perturbation’s impact is scored and ranked based on its ability to shift the diseased cells closer to a healthier state (Fig. 1e).

### Staging samples based on tissue involvement as measured by the surface density allows assessments of mesenchymal cellular population dynamics during disease progression

A true longitudinal profiling of the lung cells from the same patient across different IPF stages is impossible because patients are rarely if ever biopsied more than once. Thus, to investigate the cellular dynamics along the progression of human IPF tissues, we analyze samples from differentially affected regions of the IPF lung using a strategy previously described^7^. To build the surrogate “longitudinal” single-cell data, here we employed a Gaussian density estimator to classify all samples (and thus all cells) into different IPF stages. The model will learn the best number of IPF stages and the associated Gaussian parameters (mean and standard deviation) for each IPF tissue involvement stage based on the profiled alveolar surface density (Supplementary Fig. 1a,b) as previously described^7^. All the samples were categorized based on their alveolar surface density into 4 IPF stages: Healthy (Control, or stage 0), Normal looking IPF (stage 1), moderately involved IPF (stage 2), and Severe (stage 3). This 4-stage classification matches the existing understanding of the disease and has been previously validated^7,39–41^. After the density estimation analysis, we got the samples and cells assigned to these 4 IPF stages (Supplementary Fig. 1c). Specifically, 30 samples were categorized as healthy (135,536 cells). Seven samples were classified as the IPF stage 1 (41, 957 cells). The stage 2 data is composed of 7 samples (21, 531 cells) while the stage 3 data comprises 10 samples (22, 520 cells) (Supplementary Fig. 1d). As shown in Supplementary Fig. 1e, there’s a discernible increase in mesenchymal cells starting from IPF stage 1, hinting at a possible rise in fibroblast cells from this stage onward.

### UNAGI effectively identifies varying cell populations across IPF stages

**Fig. 2 |:**
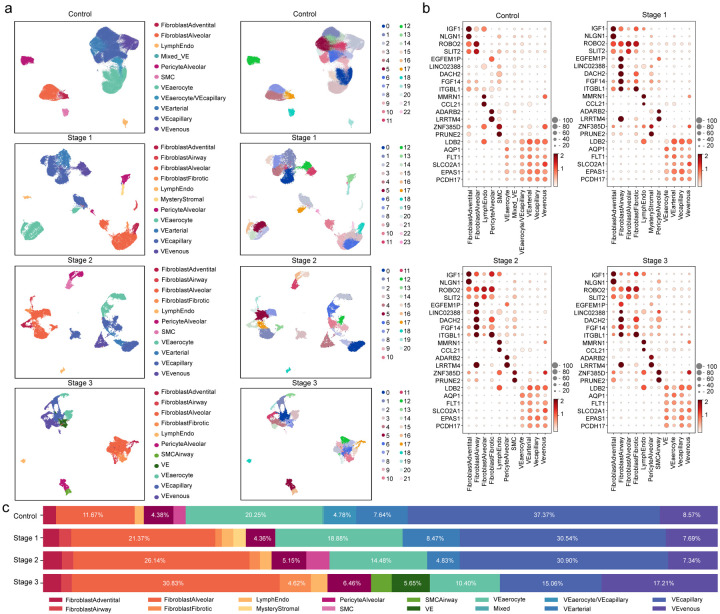
UNAGI identifies progressive heterogenous cell populations across IPF stages. **a,** UMAP visualization: Mesenchymal cells across various IPF stages are depicted. Each point corresponds to a cell; the first column categorizes them by cell type (e.g., SMC = smooth muscle cell, VE = vascular endothelial), and the second by Leiden cluster IDs. This panel underscores UNAGI’s ability to learn a potent cell embedding, ensuring premium cell clustering. **b,** Gene dot plots: Dot plots illustrating the key biomarkers for each identified cell type across four stages of IPF. In these plots, the size of each circle indicates the proportion of cells expressing the gene, and the circle’s color reflects the level of normalized gene expression. **c,** Cell composition chart: A visualization of the shifts in cell type composition along with IPF disease progression. Colors indicate the specific cell type. Notably, there’s a discernible expansion of fibroblast cells as the disease progresses.

After applying UNAGI on the four stages IPF snRNA-seq dataset and performing clustering and visualization on the latent space, we have observed a continuous trajectory of healthy fibroblasts towards corresponding, fibrotic IPF archetypes^35^, prompting us to focus on and explore stromal cells using UNAGI. UNAGI showed its effectiveness in cell embeddings by achieving a 0.74 average ARI of all stages. UNAGI identified 11 distinct cell types in Controls, with more emerging in subsequent IPF stages (Fig. 2a), which we annotated based on the expression of canonical cell markers (Fig. 2b and independent manual cell-type annotations in supplementary Fig. 2). UNAGI can capture cell subpopulations, like fibrotic fibroblasts and airway fibroblast cells, suggesting extended fibrosis through the progression. Furthermore, UNAGI revealed differences in cellular heterogeneity: Smooth muscle cells (SMC; marked by ZNF385D and PRUNE2), and alveolar pericyte cells (characterized by ADARB2 and LRRTM4) were predominantly homogenous. In contrast, fibroblast cell populations displayed greater heterogeneity, within both alveolar (denoted by ROBO2 and SLIT2^42^) and adventitial fibroblasts. Fibroblast proportions substantially increase in IPF compared to controls —from less than 15% to more than 40%—validating that fibroblast accumulation is a hallmark of IPF progression^43^ (Fig. 2c). The alveolar fibroblast cell population exhibited the most substantial increase, while the fibrotic fibroblast archetype appeared only in subsequent IPF stages. The proportions of vascular endothelial cells consistently decreased as IPF progressed. The cell embeddings from IPF data reveal a progressive cell population across IPF stages, serving as a foundation for constructing a temporal dynamic graph depicting IPF progression.

### UNAGI reconstructs the temporal dynamics graph and the underlying gene regulatory networks during disease progression

**Fig. 3 |:**
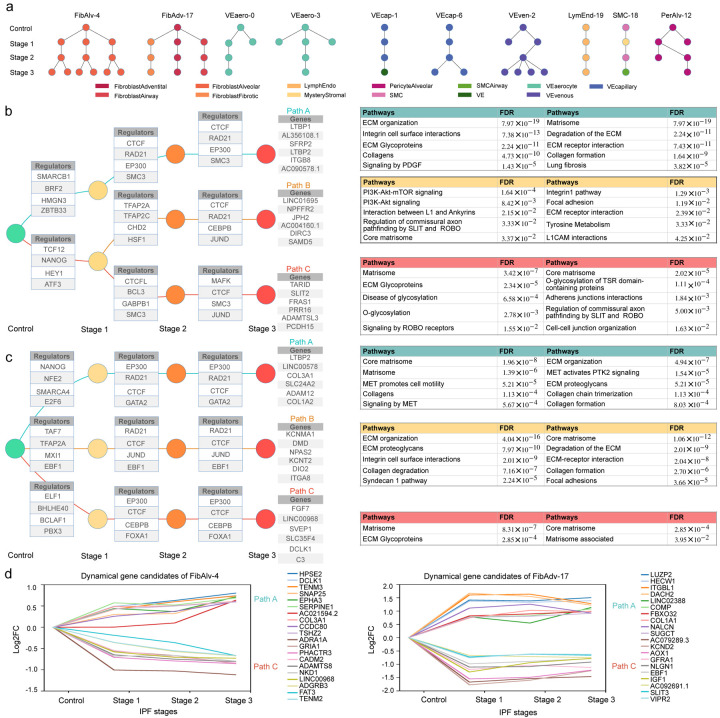
UNAGI reconstructs the temporal dynamics and the underlying gene regulatory networks of cellular dynamics during IPF progression. **a,** Dynamics graph of IPF progression within the mesenchymal cell lineage, comprising four IPF stages. Each node symbolizes a cell population, colored according to cell type, and the edges between two nodes depict the progression trajectory across disease stages. Trajectories, spanning from Control to Stage 3, are termed progression tracks. Each track is named with the specific cell type and the corresponding Control cluster-ID. **b,** Gene regulatory networks for the FibAlv-4 track, were reconstructed using the iDREM tool. Individual nodes signify a set of genes, and edges connecting two nodes represent gene regulators regulating expression changes. Paths encompassing nodes from Control to Stage 3 depict a consistent set of genes displaying the same expression changes throughout IPF progression. The enriched pathways associated with gene paths were also provided. **c,** the temporal regulatory networks for the FibAdv-17 track. **d,** Line chart of expression of the top dynamic gene candidates on the FibAlv-4 and FibAdv-17 tracks, the top 10 most increasing and the top 10 most decreasing candidate marker genes through the IPF progression.

UNAGI can effectively reconstruct the cellular dynamics associated with time series or disease progression data based on the cell embeddings learned by the model. Within our analytical framework, a “track” delineates a distinct trajectory within the reconstructed dynamics graph, marking the sequential cellular state transitions corresponding to specific cell clusters or populations. These tracks not only identify pathways but also chronicle the journey of cellular progression and evolution. Within mesenchymal cells, we have discerned 10 distinct progression tracks (Fig. 3a), transitioning from the IPF stage 0 (healthy controls) to IPF stage 3 (severe fibrosis). Two of these tracks are composed of fibroblast cells, FibAlv-4 traces the cellular state shifts of alveolar fibroblast cells during IPF progression while FibAdv-17 illustrates the cellular dynamics of remaining adventitial, airway, and fibrotic fibroblasts. Of note, the fibroblast tracks in the dynamics graph contain multiple branches, potentially reflecting the multifaceted roles of fibroblast cells in the fibrosis process^44^.

The gene regulatory network of FibAlv-4, as reconstructed by UNAGI, highlights the central role of gene regulators CTCF, RAD21, SMC3, and especially fibrosis-promoting EP300^45,46^. This is further supported by the genes in Path A of the FibAlv-4 track, which include recognized fibrosis biomarkers like LTBP1 and LTBP2^47,48^ (Fig. 3b). Pathways enriched in track FibAlv-4 include: in Path A, Collagen and Extracellular Matrix (ECM) pathways^49^; in Path B, the PI3K-Akt-mTOR signaling pathway and the focal adhesion pathway, both hallmarks of IPF fibroblasts^50–52^ (Fig. 3b); and in Path C, SLIT2, a known marker of IPF^42^. The FibAdv-17 track highlights the contribution of adventitial fibroblasts to matrix remodeling. The discovery of recognized collagen genes like COL3A1 and COL1A2^53,54^, are pivotal markers for IPF. Enriched pathways encompass general ECM-related pathways including ones of collagen formation, organization, trimerization, and degradation, with some variation between paths A to C (Fig. 3c). The MET-activated PTK2 signaling pathway^55^, a substantial player in pulmonary fibrosis progression, is also highlighted. The genes in Path B, including KCNMA1^56^, NPAS2^57^, ITGA8^58^, and DIO2^59^, all closely associated with IPF.

The depth and precision of the reconstructed gene regulatory network are underscored by its ability to pinpoint not only pivotal gene regulators and pathways but also the target genes they regulate. These target genes, especially those that exhibit differential expression across disease stages, provide invaluable insights into the temporal dynamics of IPF progression. In the context of the FibAlv-4 track, the method identifies genes like COL3A1 and SERPINE1, which are induced by the TGF-Beta pathway^60^, a key player in IPF. Furthermore, the inclusion of top dynamic marker candidates such as DCLK1^62^, TENM3, TENM2, ADRA1A, and GRIA1, all of which have established associations with IPF^61–64^, attests to the method’s robustness in capturing disease-relevant genes (Fig. 3d).

Taken, together, UNAGI’s comprehensive mapping of gene regulators, pathways, and their target genes in the reconstructed gene regulatory network underscores the method’s unparalleled capability in unraveling the intricate molecular interplay underlying IPF.

### UNAGI comprehensively captures novel dynamical and hierarchical static markers across various disease stages

Conventional single-cell analysis primarily identifies differentially expressed markers between healthy and diseased cells. In contrast, we developed UNAGI to identify dynamic marker genes that offer a longitudinal profile of cellular state changes throughout IPF progression. It discerns dynamic markers for individual cell populations, tracing gene expression shifts across disease stages. All identified candidate biomarker genes from the above cell dynamics gene regulatory network for each track will be subjected to a permutation test to assess their statistical significance. This test involves random shuffling of cells from the track across various stages. Subsequently, we calculate the sum of fold changes in gene expression between these stages to establish a background distribution for comparative analysis. Candidate genes that are deemed statistically significant through this test will be considered as dynamic markers, closely associated with the track in the analysis (as detailed in the “Dynamic markers discovery” section of the Methods).

**Fig. 4 |:**
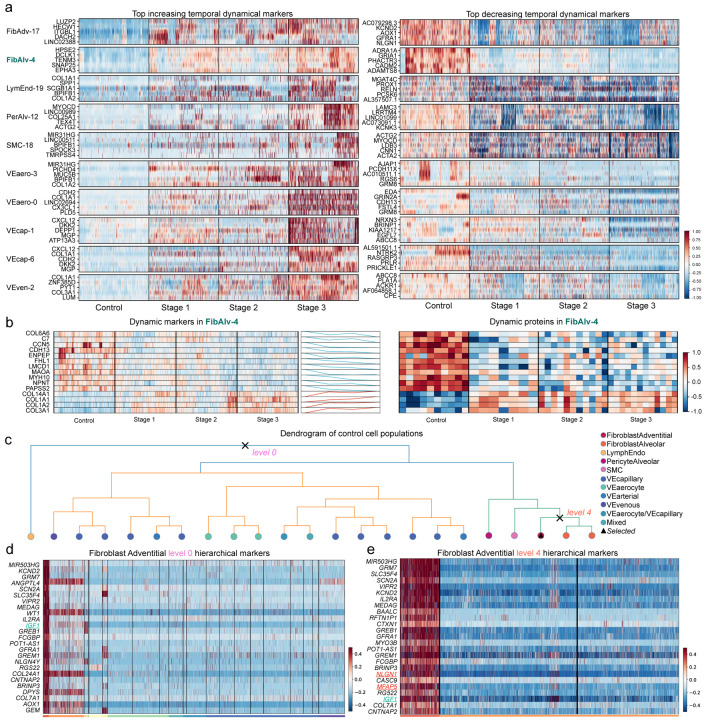
UNAGI comprehensively captures novel dynamical and hierarchical static markers across various IPF stages. **a,** Heatmaps presenting the most pronounced increasing (left) and decreasing (right) temporal dynamic markers’ expressions, each z-score normalized, across tracks. **b,** The left panel showcases heatmaps of dynamic gene markers from the FibAlv-4 cluster. Importantly, the right panel provides experimental verification of these markers through corresponding protein expressions derived from proteomics data. Line plots accompanying these highlight gene expression shifts of these dynamic markers over the course of IPF progression. **c,** Dendrogram visualizing control cell populations. Each node signifies a cell type-specific population. The Fibroblast Adventitial cluster is accentuated. Using UNAGI, various hierarchical biomarkers are discernible at different levels, either contrasting with other cell types or juxtaposing subpopulations within the same cell type. **d,** Heatmap detailing the top 25 hierarchical static markers’ expressions, all z-score normalized, for the Fibroblast Adventitial cluster at level 0. This highlights UNAGI’s proficiency in pinpointing general cell type markers. **e,** Heatmap delineating the top 25 hierarchical marker gene expressions, z-score normalized, for the Fibroblast Adventitial cluster at level 4, set against two Fibroblast Alveolar clusters, emphasizing UNAGI’s capability in cell subtype marker identification.

Fig. 4a showcases heatmaps of the top 5 dynamic markers for each track, both those that increase and decrease during disease progression (a comprehensive list is available in Supplementary Table 1). For instance, in the FibAdv-17 track, markers like LUZP2, ITGBL1, and AOX1, previously reported as differentially expressed in IPF^65^, are highlighted. Notably, NLGN1, GFRA1, and AOX1 are markers for adventitial fibroblasts^66^ and emerge as a top-decreasing temporal dynamic marker in this track, suggestive of a loss of respective cell identity. The FibAlv-4 track, on the other hand, features markers like DCLK1, TENM3, ADRA1A, GRIA1, and EPHA3, all of which have strong ties to lung fibrosis^61–64,67^. It is important to mention that while our discussion here primarily focused on monotonically increasing and decreasing biomarkers, which are of main interest in our study, the model we developed is also able to identify biomarker genes with other patterns. An example of this is genes that initially increase and then decrease, as observed in path B of the FibAdv-17 track.

To experimentally verify the dynamic markers identified by UNAGI, we performed proteomics of matched tissue blocks, 3 samples each from 10 IPF patients across different stages (based on the same surface density criteria) and one each from 10 control donors (Supplementary Table 2). We identified 1070 dynamic proteins from the proteomics data and 606 dynamic gene markers in the snRNA-seq dataset. Further analysis revealed that 151 dynamic proteins have corresponding protein-coding genes in the snRNA-seq data. Interestingly, 40 out of 151 dynamic markers overlapped with dynamic proteins(Supplementary Fig. 3a). Hypergeometric testing on individual tracks revealed statistical significance (*P* – *value* < 0.05 is set as the default cut-off) for protein-coding genes of dynamic proteins in four specific tracks: FibAlv-4 (*P* – *value* = 0.003), VEven-2 (*P* – *value* = 0.015), LymEnd-19 (*P* – *value* = 0.033), and VEcap-1 (*P* − *value* = 0.048), as well as in the overall result (Supplementary Fig. 3b). Notably, the FibAlv-4 track contained 137 dynamic protein-encoding genes, and 14 of these genes produce dynamic proteins (Fig 4b).

A notable observation from our snRNA-seq and proteomics data is that five of these overlapping dynamic markers are collagens (COL1A1, COL1A2, COL3A1, COL3A1, COL14A1), confirming that progressive matrix remodeling is intrinsically intertwined with the development of fibrosis^68^. Moreover, a majority of the overlapped dynamic markers have been previously associated with pulmonary fibrosis^54,69–71^, including CCN5, CDH13, MYH10, PAPSS2, and LMCD. This proteomics data serves as a validation, confirming that the discovered dynamical genes play a crucial role in the progression of IPF.

UNAGI can identify both dynamic and static markers. While dynamic markers offer insights into cellular state changes throughout disease progression, static markers are crucial for distinguishing between different cell types and subpopulations within a given stage. Existing static biomarker discovery pipelines^11,12^ usually employ a “one vs. the rest” strategy and may fail to distinguish the difference between different subtypes.

UNAGI explores the hierarchies of marker genes that not only distinguish different cell populations but also capture the finer heterogeneity among cell subpopulations. For instance, focusing on the FibAdv-17 cluster of controls, cell subpopulations are primarily divided into three main groups: fibroblasts, vascular endothelial cells, and lymphatic endothelial cells (Fig. 4c, and dendrograms of all four stages are in Supplementary Fig. 4). The fibroblast adventitial population spans 5 levels in the dendrogram. Fig. 4d showcases the top twenty-five positive hierarchical static markers for fibroblast adventitial cells at dendrogram level 0. These markers distinguish the fibroblast adventitial cluster from all other clusters. UNAGI’s results are consistent with the dendrogram structure, highlighting the close relationship between fibroblast adventitial and fibroblast alveolar clusters. Notably, UNAGI identified key markers like IGF1 and collagen genes such as COL24A1 and COL7A1, emphasizing the role of elevated interstitial collagen levels in IPF^72^. Other markers like ANGPTL4 and WT1 further underscore the method’s precision in identifying relevant genes^73^ (top 25 level 0 positive and negative markers are detailed in Supplementary Fig. 5).

Fig. 4e presents the top 25 positive hierarchical static markers for the fibroblast adventitial cluster at level 4 (sub-type level). While there’s an overlap with level 0 markers, level 4 introduces unique markers potentially for subtypes like NLGN1, a cell type marker for adventitial fibroblastas^66^, and MFAP5, previous research indicates that MFAP5+ fibroblast cells are localized to vascular adventitia^74^ (top 25 level 4 positive and negative markers are detailed in Supplementary Fig. 6). UNAGI’s ability to identify both temporal dynamic markers and hierarchical static markers offers a comprehensive lens to study IPF. This dual approach allows for detailed profiling of the disease from both intra-stage and longitudinal (inter-stage) perspectives, enhancing our understanding of its complexities.

### UNAGI identifies potential therapeutic pathways for IPF treatments

In the preceding sections, we delved into how UNAGI enhances our comprehension of biomarkers and cellular dynamics in the progression of IPF. Building upon this foundational understanding, we now shift our focus to the therapeutic frontiers opened by UNAGI. This involves leveraging its in-silico perturbation capabilities, which are rooted in IPF-specific cell embeddings and the temporal dynamic graph of IPF. This approach paves the way for pinpointing potential therapeutic targets and pathways, a development that promises to be a substantial stride in IPF treatment. Detailed results of these pathway perturbations are systematically presented in Supplementary Table 3.

Many of the top pathways predicted by UNAGI are in alignment with known IPF-centric pathways. Impressively, of the top 10 therapeutic pathways identified, at least seven have been previously associated with IPF progression and potential treatments. For instance, the discovery of the ROBO pathway: Signaling by ROBO receptors (Score=0.5890, FDR= 1.1028 × 10^−14^) which has been strongly linked to IPF progression^42,75^. Additionally, the Netrin 1 signaling pathway (Score=0.6548, FDR= 3.4698 × 10^−19^) has been correlated with various forms of pulmonary fibrosis, including bleomycin-induced pulmonary fibrosis^62,76^. Moreover, UNAGI’s ability to identify target genes within these pathways offers a deeper understanding of disease progression. For example, the Calcium signaling pathway in fibroblast is highly related to fibrosis^77^, genes within the pathway such as EGFR, which is linked to fibroblast proliferation^77,78^, have been highlighted. Beyond known pathways, UNAGI also uncovered novel pathways. While some of these pathways might not have a direct association with IPF, their target genes often play critical roles in the disease’s progression. This is evident in the discovery of genes like GRIA1^61,79^ and ERBB4^63^, both of which have substantial associations with IPF progression.

**Fig. 5 |:**
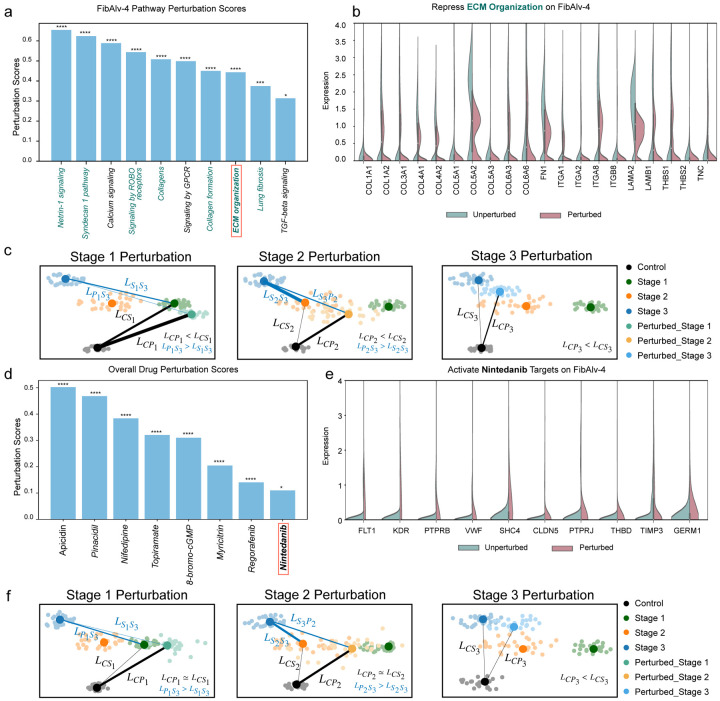
UNAGI identifies potential therapeutic pathways and potent drugs for IPF treatments. **a,** Bar chart of the track FibAlv-4 pathway perturbation results. The highlighted pathways are also identified in the reconstructed gene regulatory network of the track. **b,** Split-violin plot of the gene expression differences for the top 20 most changing genes of in-silico extracellular matrix (ECM) organization pathway perturbation in Stage 1 of the FibAlv-4 track. **c,** PCA plots of latent space Z of in-silico ECM organization pathway perturbation effects and dots represent cells from distinct stages. Lines connected to two nodes are the PAGA connectivity score between two clusters, where the width of a line is proportional to the strength of the score, and the length of the line can represent the distance between the UNAGI embeddings of the two connected clusters. (e.g., Line connecting Control and Perturbed Stage 1 $\left(L_{CP_{1}}\right)$). **d,** Bar chart of the top overall drug perturbation results. **e,** Split-violin plot of gene expressions for the top 10 changing targets of Nintedanib in the gene interactions network both before and after perturbation in Stage 1 of the FibAlv-4 track. **f,** PCA plots of Nintedanib perturbation effectiveness.

The results from pathway perturbations, as showcased in Fig. 5a,b, further demonstrate UNAGI’s capability. The perturbations reveal the potential of certain pathways to revert cells to a healthier state, suggesting their therapeutic potential. For instance, the perturbation of the ECM organization pathway in various IPF stages suggests its potential to guide cells to a less severe IPF cellular state.

A central takeaway from Fig. 5c is its demonstration that *in-silico* pathway perturbations effectively shift the cellular states towards healthier conditions. The perturbation’s impact on gene expression, especially within the ECM organization pathway, is evident in the reduction of regulated gene expressions. By introducing these perturbed cells into the VAE-GAN model, cell embeddings are produced. To visualize these embeddings, Principal Components Analysis (PCA) was employed. Fig. 5c provides a visual representation of the effects of repressing the ECM organization pathway across IPF stages 1, 2, and 3. In the Stage 1 perturbation, the perturbed cell population, denoted as *P*_1_, is observed to be closer to the Control stage C than to the stage 1 cells, *S*_1_, and is notably distant from stage 3 cells, *S*_3_. The distance in the PCA plot serves as a metric of similarity, indicating that *P*_1_ more closely resembles the Control stage C than *S*_1_, suggesting a potential regression of the cellular state towards a healthier condition. This similarity is further emphasized by the thickness of connection lines between cell clusters, which represents the strength of the PAGA connectivity score. Specifically, the line $L_{CP_{1}}$ is thicker than $L_{CS_{1}}$, indicating a higher similarity between the Control stage C and *P*_1_ in the latent space. In the Stage 2 perturbation, the perturbed cell population, *P*_2_, gravitates towards *S*_1_. Both PCA distance and PAGA connectivity scores suggest that a perturbation in Stage 2 could potentially guide cells towards a milder IPF cellular state. A similar trend is observed in Stage 3, where the perturbed cell population, *P*_3_, shifts towards a relatively healthier cellular state.

### UNAGI discovers novel drug candidates for IPF treatments

UNAGI’s *in-silico* drug perturbation approach, akin to its pathway perturbation, leverages and integrates the CMAP dataset. Hereby, UNAGI has pinpointed several drug candidates that could substantially impact the progression of IPF (Fig. 5d). Comprehensive results of all drug perturbations are detailed in Supplementary Table 4.

**Apicidin**, with a score of 0.5021 and an FDR=4.551 × 10^−105^, is a histone deacetylase inhibitor. Previous studies have highlighted its potential as an antifibrotic drug in pulmonary fibrosis^80,81^. **Nifedipine**, scoring 0.3834 with an FDR= 1.152 × 10^−57^, has been shown to reduce bleomycin-induced pulmonary fibrosis by disrupting calcium signaling in fibroblasts^78^. **Cilomilast**, a phosphodiesterase 4 (PDE4) inhibitor, has a score of 0.3082 and an FDR= 4.407 × 10^−35^. It has demonstrated potential in attenuating pulmonary fibrosis in mice^82^. **Niguldipine**, scoring 0.3842 and an FDR= 6.160 × 10^−58^, is a calcium channel blocker and an α1-adrenergic receptor antagonist, showing anti-fibrotic effects in the lung^78^. The compound **8-bromo-cGMP**, which impacts PRKG1, has a score of 0.3099 and an FDR= 1.562 × 10^−35^, and is associated with the TGF-beta pathways in the fibrosis process^83^.

Moreover, drugs like **Ibudilast** (Score=0.3053, FDR= 2.465 × 10^−34^) and **Topiramate** (Score=0.3203, FDR= 2.411 × 10^−38^) have been identified, with the former potentially having anti-fibrotic effects similar to other PDE4 inhibitors^84^, and the latter regulating GRIA1, which is associated with lung fibrotic diseases^61,79^. **Myricitrin** (Score=0.2045, FDR=2.590 × 10^−13^) has been shown to exhibit anti-fibrotic activity in certain conditions^85^, while **Regorafenib** (Score= 0.1407, FDR= 2.653 × 10^−5^)) attenuates fibrosis by inhibiting the TGF-beta pathway^86^. Notably, UNAGI also identified **Nintedanib** (Score=0.1102, FDR=0.0111), an FDA-approved drug for IPF treatment.

The target gene intervention of Nintedanib is shown in Fig. 5e. The corresponding perturbation results, visualized in Fig. 5f across IPF stages 1, 2, and 3, emphasize the potential of these drugs to shift cell populations towards healthier states. The consistently higher PAGA connectivity scores between perturbed cell populations and healthier cellular stages indicate that the perturbed cell populations are more akin to healthier cells.

### Benchmarking and computational verifications

**Fig. 6 |:**
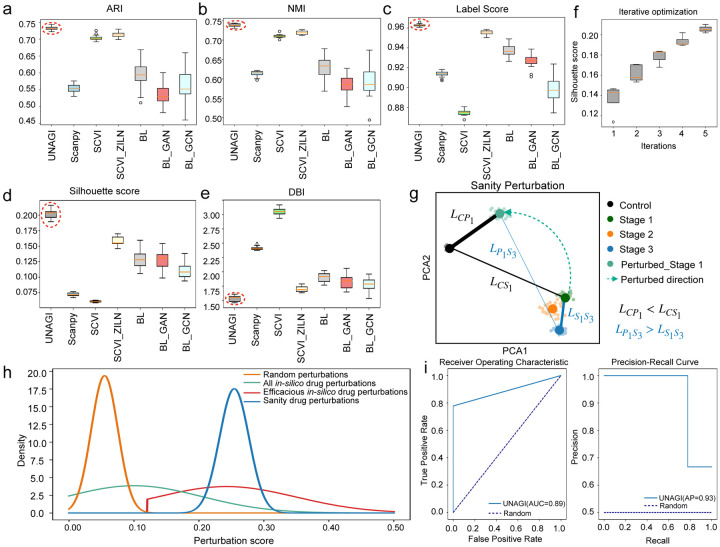
UNAGI outperforms alternative approaches in learning the cell embeddings and can effectively identify efficacious drugs in *in-*silico perturbations. **a,** Adjusted Rand Index (ARI) and **b,** Normalized Mutual Information (NMI) illustrate the effectiveness of the learned cell embeddings for downstream clustering tasks. **c,** Label score, indicating that cells within neighborhoods primarily have the same cell type. **d,** Silhouette score. **e,** Davis-Bouldin index (DBI); a lower DBI signifies better clustering. These scores (**c, d, e**) are unsupervised metrics employed to demonstrate the clustering quality derived from the learned cell embeddings. **f,** Box plot presenting the silhouette scores of UNAGI across various training iterations, emphasizing that the iterative strategy progressively enhances cell embeddings and clustering quality with each iteration. **g,** PCA representation highlighting the impact of sanitary perturbation, which involves reversing the gene expression at Stage 1 back to the patterns observed in the control stage. This process essentially seeks to “normalize” or “sanitize” the aberrant gene expressions, bringing them in line with a control or reference state. **h,** Distribution patterns for various drug/compound perturbations. The x-axis represents the perturbation score, while the y-axis portrays the density of the fitted Gaussian distribution for each specific setting. **i,** AUROC and AUPRC metrics in relation to perturbation verification. As a reference, a random drug effectiveness predictor is used as a baseline, with an AUC (Area Under the Curve) score of 0.5, indicating no predictive discrimination, and an average precision (AP) score of 0.5, representing a baseline precision level.

To underscore the effectiveness of UNAGI in cell representation learning, we juxtaposed its capabilities against established methods like scVI^15^ and SCANPY^12^. scVI, a deep generative model rooted in VAE, employs the zero-inflated negative binomial (ZINB) distribution to capture the raw count distribution of single-cell data. On the other hand, SCANPY, a standard single-cell analysis pipeline, relies on PCA to encapsulate the high-dimensional single-cell data. Moreover, we performed ablation experiments to verify the effectiveness of individual computational module components of UNAGI. The UNAGI framework’s VAE model is designed with the flexibility to select from various data distributions, such as Gaussian, NB (Negative Binomial), ZINB, and ZILN depending on the observed gene expression distribution in a given dataset. For the IPF snRNA-seq data analyzed in this study, the gene expression distribution aligns with a ZILN pattern. To showcase the benefits of adopting a proper data distribution to the cell embedding learning, the VAE model employing ZILN distribution served as the baseline model (BL), GAN and GCN were added to the BL model in our UNAGI framework. Contrasting with the ablation baseline, the full-fledged UNAGI framework integrates all the key components — the ZILN, GAN, and GCN — for the effective learning of cell embeddings. To ensure a balanced evaluation, we employed the Leiden clustering method on the embeddings generated by all seven techniques, adjusting parameters to produce a comparable number of clusters for each stage (UMAPs of all benchmarking methods are detailed in Supplementary Fig. 7). Five metrics were evaluated (Fig. 6a–e): For label metrics including Adjust Rand Index (ARI)^87^ and Nearest Mutual Information (NMI)^88^, UNAGI steadily outperforms SCANPY and scVI. For non-label metrics, the label score^89^ of UNAGI is at least 4% better than other methods, also suggesting a high homogeneity inside cell neighborhoods. With an average silhouette score nearing 0.205, UNAGI’s clusters are more cohesive and distinct. The Davies-Bouldin Index (DBI), averaging at a lower 1.6, further attests to UNAGI’s ability to produce clusters that are not only distinct but also internally homogeneous. Diving deeper, we explored the potential of the ZILN distribution to better model normalized single-cell data compared to the ZINB in this study. When scVI was adapted to this distribution, its performance surpassed its original ZINB-based counterpart in this dataset. This underscores the ZILN model’s proficiency in capturing the nuanced continuous information inherent in normalized single-cell data, as opposed to the more discrete ZINB. The silhouette score presented in Fig. 6f effectively illustrates the benefits of iterative optimization (additional metrics refer to Supplementary Fig. 8). This result underscores the importance of the iterative training approach, which fosters a synergistic relationship between cell-embedding learning and the inference of cellular dynamics. Such an approach not only markedly enhances the method’s performance over successive iterations but also demonstrates that disease-sensitive cell representation learning is instrumental in achieving improved cell clustering and potentially enhancing other downstream tasks.

To rigorously evaluate the proficiency of UNAGI’s in-silico perturbations, we embarked on a Stage 1 sanity perturbation, wherein the perturbations were aimed at redirecting them back to control levels (refer to methods for an in-depth explanation). Cells subjected to sanity perturbation overwhelmingly align with the control stage as opposed to their native Stage 1 state (Fig. 6g). Reinforcing this trend, the PAGA connectivity scores indicate that these perturbed cells bear a high resemblance to Control cells, and they substantially deviate from Stage 3 cells. These observations strongly vouch for UNAGI’s unmatched precision and efficacy in handling in-silico perturbations. In addition, we delved deeper into assessing UNAGI’s aptitude for identifying potent drug candidates using in-silico methodologies. The insights, presented in Fig. 6h, underscore the salience of this tool: in-silico drug perturbations, particularly those with significant FDR values, consistently surpassed the therapeutic scores of random perturbations as expected. Impressively, these results were congruent with the outcomes from sanity drug perturbations, during which we intentionally tweaked target gene expressions to reflect that of an adjacent, healthier stage. This robust alignment unequivocally attests to UNAGI’s exceptional ability to single out drugs with a high potential to combat IPF.

To further stress-test UNAGI’s capabilities, especially its prowess in drug repurposing, we set forth a meticulously planned simulation. This simulation was designed to test UNAGI’s ability to accurately identify and replicate the effects of known drugs, referred to as ‘ground-truth’ drugs in our in-silico drug discovery simulation (as detailed in the Methods section). Our approach involved a strategic implantation of drugs within the simulation. We did this by altering the target gene expressions of these drugs at specific magnitudes across various disease stages, thereby establishing a set of ground truths. These modified gene expression levels were intended to mimic the real-world effects of the drugs at different stages of disease progression. Next, we applied the UNAGI to the simulated dataset (with ground truth) to predict the drugs that act against the simulated gene expression changes and examine whether the model can recapture the implanted drugs in the simulation data. The performance of UNAGI in this simulation was rigorously evaluated using two established metrics: the Area Under the Receiver Operating Characteristic Curve (AUROC) and the Area Under the Precision-Recall Curve (AUPRC). UNAGI achieves scores of 0.89 and 0.93, respectively (Fig. 6i). These high scores indicate that UNAGI is highly effective at identifying drugs that target genes with dynamic regulation during disease progression.

### Experimental validation of in-silico drug perturbations via precision-cut lung slices.

**Fig. 7 |:**
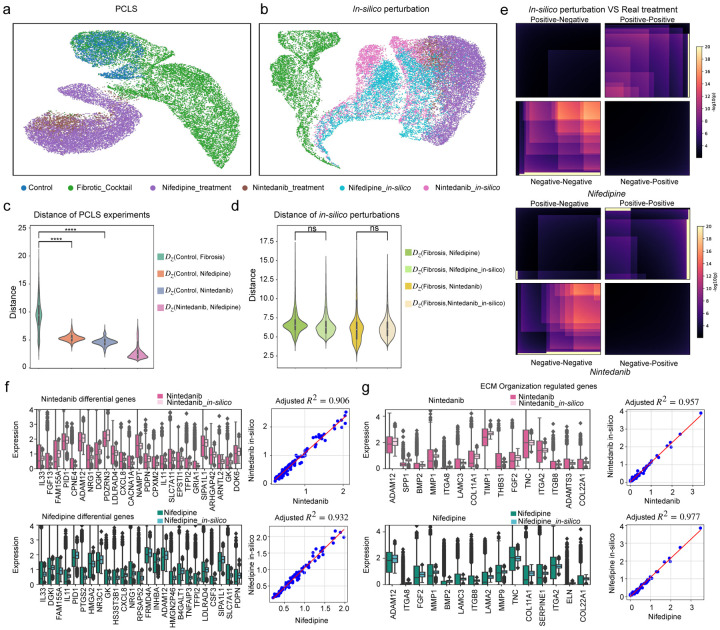
The predictions of UNAGI align with human precision-cut lung slices (PCLS) drug validations. **a,** UMAP visualization of the PCLS data with each dot representing an individual cell. **b,** UMAP representation emphasizing the similarity between the real-world treatments of Nifedipine and Nintedanib. Furthermore, cells under in-silico drug treatments (Nifedipine and Nintedanib) closely mirror those under actual treatments. **c,** Violin plots showcasing that Nintedanib and Nifedipine treatments markedly shift fibrotic cells, bringing them closer in resemblance to healthy control cells. (e.g., *D*_*z*_(Fibrosis, Nifedipine) is the distance between fibrosis cells and fibrosis cells after Nifedipine treatment). **d,** Violin plots highlighting the strong alignment between in-silico drug treatments and their real-world counterparts. **e,** The RRHO (Ranked Rank Hypergeometric Overlap) plots for both Nifedipine and Nintedanib. These plots juxtapose in-silico perturbations post-VAE reconstruction against actual treatments, emphasizing the high degree of similarity between in-silico and real treatments. Specifically, the genes up-regulated and down-regulated in in-silico treatments show strong correlations with those affected in real treatments. **f,** Box plots and *R*^2^ plots compare the expression of the top differential genes of real treatments (Nintedanib or Nifedipine vs. fibrosis) to in-silico perturbation results. The box plot visualizes the top 25 differential genes (ranked based on log fold changes) for each treatment. The gene expression of the top 100 differential genes in real and in-silico drug treatments are used to calculate the adjusted *R*^2^ metric and generate *R*^2^ plots. This representation is intended to underline the remarkable similarity observed between in-silico drug perturbations and the corresponding actual drug treatments for both Nifedipine and Nintedanib. **g**, Box plots and *R*^2^ plots of ECM organization target gene expressions from real treatments and in-silico perturbations. The box plots visualize the top 15 genes of ECM based on log fold changes between real treatments and fibrosis cells. The gene expressions of all ECM organization target genes in real and in-silico drug treatments are used to calculate the adjusted *R*^2^ metric and generate *R*^2^ plots.

To experimentally validate UNAGI predictions, we utilized the fibrotic cocktail model of PCLS^90^. We chose to test the model predictions for Nifedipine, an antihypertensive drug not known to have a role in fibrosis treatment, and Nintedanib, an FDA-approved drug for IPF. We stimulated PCLS with DMSO as the Control for 5 days. In the treatment group, PCLS were treated for 3 days with the fibrotic cocktail to induce fibrosis, and treatment, Nifedipine or Nintedanib, started from day 3 until day 5. As read-out, we performed snRNA sequencing (Fig. 7). Latent embeddings of fibroblasts derived from this PCLS experiment reveal intriguing patterns (Fig. 7a). When assessed based on experimental conditions, cells under both Nifedipine and Nintedanib treatments exhibit similar clustering behaviors on the UMAP. This suggests their parallel roles in inhibiting fibroblast activation.

Utilizing UNAGI’s perturbation module, Nintedanib and Nifedipine in-silico perturbed cells gravitate towards the Nintedanib treated population, demonstrating potential therapeutic effects (Fig. 7b). Pairwise Euclidean distances between latent embeddings indicate that both treatments effectively steer the cellular state of fibrosis cells toward a healthier baseline (Fig. 7c) and the *in-silico* treatments behave like real treatments (Fig. 7d). This observation is solidified by the Mann-Whitney U test confirming the analogous anti-fibrotic properties of both treatments. The Ranked Rank Hypergeometric Overlap (RRHO) confirms that the markers identified in-silico align closely with the biomarkers observed in the PCLS experiments (Fig. 7e). The adjusted *R*^2^ scores for Nintedanib in-silico (0.906) and Nifedipine in-silico (0.932) with respect to the top 100 differentially expressed genes (DEGs) in actual treatment versus fibrosis, as well as the top 25 markers in side-by-side comparisons (Fig. 7f, top 100 DEGs comparisons are detailed in Supplementary Fig. 9), demonstrate the consistency of gene expression patterns between in-silico and real treatments markers. Known IPF markers like IL33^91^, ADAM12^92^, and CXCL8^93^ exhibit similar changes in gene expression in both real treatment experiments and in-silico predictions. (Fig. 7g, all ECM organization pathway genes comparisons are listed in Supplementary Fig. 10). The *R*^2^ scores and side-by-side comparisons of real treatments and in-silico gene expression of the ECM organization pathway further validate the capability of the UNAGI model to accurately simulate in-silico perturbations on IPF-related targets. The alignment between in-silico drug perturbations and actual drug treatments on the PCLS stands as a testament to the reliability of UNAGI’s predictions.

### UNAGI *in-silico* analysis unveils COVID-19 cellular dynamics and therapeutic opportunities

To demonstrate the expansive applicability of UNAGI to various complex diseases, we studied the intricate dynamics of COVID-19. We used a subset of a COVID-19 dataset^94^ consisting of 246,948 peripheral blood mononuclear cells from 130 samples with various severities of COVID-19. We categorized them into four COVID-19 stages based on the disease severity of patients: Healthy (Control, or stage 0), Asymptomatic or Mild (stage 1), Moderate (stage 2), and Severe or Critical (stage 3). The UNAGI pipeline is applied to the COVID-19 dataset to reveal the temporal dynamics and discover potential therapeutic targets.

**Fig. 8 |:**
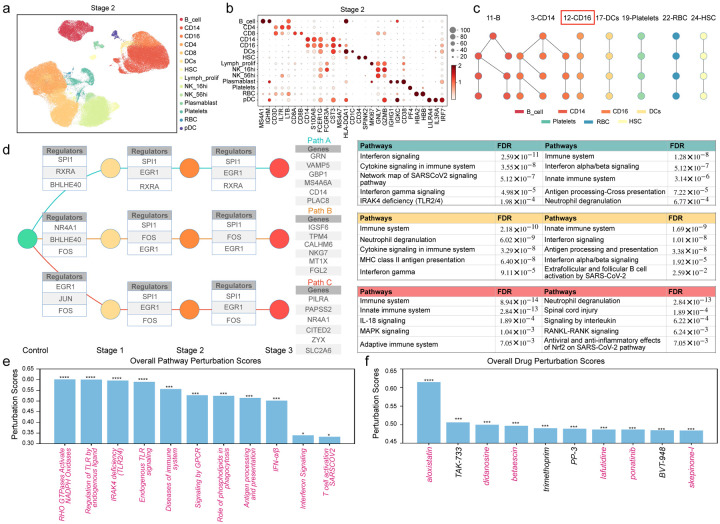
UNAGI *in-silico* analysis unveils COVID-19 cellular dynamics and therapeutic opportunities. **a,** UMAP display of stage 2 COVID-19 data with each dot symbolizing an individual cell. Cells are color-coded based on their respective cell types. **b,** Dot plot illustrating the expression levels of canonical cell type markers present within the stage 2 COVID-19 data set. **c,** Dynamic graphs representing the cellular dynamics underlying the COVID-19 progression. Within these graphs, each node corresponds to a cell cluster, and the connecting edges signify the relationships between these nodes (shift of the cell population along with COVID-19 progression). **d,** Depiction of the reconstructed gene regulatory network for the track 12-CD16. Prominent gene regulators, genes, and pathways discerned from the enrichment analysis are enumerated. **e,** Bar chart detailing the principal pathway perturbation outcomes. Pathways highlighted have literature support, indicating their potential as therapeutic targets against COVID-19. **f,** Bar chart outlining the top 10 drug perturbation results. Drugs that are emphasized have been highlighted based on literature support, suggesting their candidacy for treating COVID-19.

After learning the latent cell representations (Supplementary Fig. 11), UNAGI identified 14 unique cell populations at stage 2 (Fig. 8a), spotlighting nuanced interactions such as between platelet and T-cells, a finding resonating with the previous research^94^. Here, UNAGI can elucidate unique markers for cell populations, such as MS4A1 and CD79A in B cells, and underscore differential expressions, notably CD8A and CD8B, in CD8 T cells—findings that harmonize with manual annotations (Fig. 8b).

Focusing on the cellular dynamics across the trajectory of COVID-19, UNAGI identified 7 distinctive tracks reflecting the evolving cellular interplay across the disease stages (Fig. 8c). Fig. 8d deepens the narrative, spotlighting pivotal genes central to the COVID-19’s progression of CD16+ Monocytes, like BHLHE40 which finds an up-regulation in moderate patients^95^, and EGR1, recognized for influencing SARS-CoV-2 replication and antiviral responses^96^. Notably, genes like GRN^97^ and PLAC8^98^ emerge as notably up-regulated in COVID-19. Gene enrichment analyses further discern crucial pathways tied to the disease such as interferon signaling and immune system pathways^99–101^. Transitioning to predictive capabilities, UNAGI identified potential therapeutic pathways such as the RHO GTPases Activate NADPH Oxidases pathway aligns with modern findings emphasizing its substantial role in COVID-19^102,103^ (Fig. 8e). A deep dive into pathways related to Toll-Like Receptors and interferon responses^104^ further broadens the therapeutic landscape.

In culmination, Figure 8f accentuates UNAGI’s expertise in drug recommendation. Aloxistatin stands out, achieving the highest drug perturbation scores and drawing attention due to its potential against SARS-CoV-2 proteases^105^. Additionally, Didanosine, notable for its efficacy against COVID-19 Polymerase and Exonuclease^106^, and Ponatinib, are recognized as potent COVID-19 drugs by other machine learning methods^107^. This detailed alignment with ongoing research^105–109^ not only emphasizes UNAGI’s precision but heralds its indispensable role in crafting therapeutic strategies for multifaceted diseases.

## Discussion

In this manuscript, we describe UNAGI, a computational tool designed to model the temporal cellular dynamics inherent in complex disease progression. Rooted in its design is the use of a Gaussian Mixture Model-based density estimator, which classifies samples into specific disease stages. Harnessing the power of the graph VAE-GAN model, UNAGI handles high-dimensional single-cell data to extract latent embeddings. These embeddings play a crucial role in formulating progression tracks for distinct cell populations, subsequently facilitating the detailed reconstruction of temporal gene regulatory networks. The implementation of UNAGI to differentially affected tissues in IPF allows high-resolution modeling of the cellular trajectories, pivotal gene regulators, and genes that drive or associate with progressive tissue fibrosis in the human lung. Through iterative training, UNAGI sharpens the focus on IPF-specific features, priming itself for simulating and evaluating perturbations on potential target genes and drugs. The fruits of this methodical approach are manifold: UNAGI not only delivers an in-depth understanding of cellular dynamics and the foundational cell-specific gene regulatory networks during the progression of fibrosis but also pinpoints potential therapeutic pathways and drugs against IPF, marking the potential of UNAGI in modeling disease and developing novel therapeutics.

UNAGI offers a suite of characteristics that distinguish it in the domain of disease comprehension and therapeutic discovery. First and foremost, UNAGI distinguishes itself by its proficiency in creating precise cell embeddings and synthesizing cells via a deep generative neural network. This precision in embeddings enables enhanced cell clustering and identification, surpassing existing methods primarily focused on general cell representation learning. Different from other existing methods, UNAGI’s approach involves learning and incorporating key genes, including dynamic markers and gene regulators, that are integral to the specific disease progression. This results in disease-oriented cell embeddings that are finely tuned for diverse downstream tasks related to the disease. Additionally, UNAGI’s capability to artificially generate cells from the learned latent space is leveraged to conduct *in-silico* perturbations. This feature adds a dynamic aspect to disease modeling, allowing for more comprehensive and nuanced exploration of disease mechanisms and potential treatments. Third, UNAGI excels in unraveling the intricate cellular dynamics associated with the progression of a disease. Utilizing the cell embeddings it generates, UNAGI employs a graphical methodology to construct a ‘cellular dynamics tree’. This tree effectively maps out the transitions of various cell states and populations as the disease advances. Crucially, UNAGI goes a step further by identifying the underlying gene regulatory network that governs these cellular dynamics, thereby highlighting potential biomarkers and therapeutic targets. In addition, UNAGI’s sophisticated analysis enables the comprehensive identification of dynamic markers that track the evolution of the disease, as well as hierarchical static markers that differentiate between distinct cell populations. This dual approach provides a detailed understanding of the cellular heterogeneity at different stages of the disease and its transformation throughout its progression, offering valuable insights into the disease’s biology and potential intervention points. Fourth, a standout feature of UNAGI, setting it apart from existing methods, is its ability to perform unsupervised *in-silico* analysis of pathways and drug perturbations. This aspect of UNAGI allows for the exploration and identification of promising therapeutic pathways and potential drug candidates without the need for pre-existing drug perturbation training datasets, which are often challenging to acquire. This capability provides users with a powerful tool to investigate, evaluate, and prioritize therapeutic options associated with different pathway alterations or drug interventions. As a result, UNAGI uncovers a wealth of potential therapeutic strategies and promising drug candidates. Its unsupervised nature substantially enhances the method’s applicability and practicality across a variety of complex diseases, offering an advantage over many current drug perturbation approaches that rely on supervised learning and extensive training sets. Lastly, to democratize access to its innovations, we’ve launched the UNAGI web server, an interactive framework that brings cellular dynamics to life and facilitates in-silico perturbation functions, streamlining the exploration of disease dynamics and potential therapeutic interventions.

As described UNAGI has led to many biological observations, first by allowing us to uncover in an unbiased manner the trajectories that mesenchymal cells undergo during the progression of fibrosis. One notable observation is the marked proliferation of fibroblast cells, which correlates with the extensive accumulation of fibrosis, a defining characteristic of IPF progression. This proliferation underscores the fibroblasts’ central role in the disease’s pathology. Moreover, we noted that adventitial and alveolar cells exhibit dynamic and active involvement in IPF development. Conversely, the proportion of vascular endothelial cells consistently decreased as IPF progressed. Second, it allows identifying the cell-specific gene regulators that drive the phenotypic changes during disease progressions such as gene regulators like CTCF, EP300, and SMC3 and their target genes, identified dynamic markers such as COL1A1, COL1A2, COL1A3, and COL14A1, which were validated by proteomics analysis, and suggested hierarchical static markers for sub-cell types, including NLGN1 and MFAP5 for fibroblast adventitial cells. These discoveries enrich our understanding of IPF and could lead to the identification of novel biomarkers and more precise therapies. Finally, UNAGI also illuminates potential IPF pathways that could be targeted for therapeutic interventions like Netrin-1 signaling and Signaling by ROBO receptors, as well as drugs that may reverse these pathways. Impressively, we were able to validate our model’s predictions regarding drugs like nifedipine, previously not considered an antifibrotic, and potentially identify marker genes that could be used in the future as biomarkers for target engagement and efficacy. Moreover, UNAGI extends its utility to other complex diseases, such as COVID-19, with several of its top drug predictions corroborated as repurposed medications for COVID-19, including Aloxistatin and Didanosine, highlighting its broad potential in biomedical research.

Despite its array of abilities, it’s imperative to recognize UNAGI’s limitations, especially its dependency on the CMAP database for *in-silico* drug perturbation. The CMAP database, though invaluable, has its set of challenges. It doesn’t encompass all potential drugs and compounds, thereby narrowing UNAGI’s drug discovery horizon. Additionally, the impact of numerous drug perturbations on a variety of cell types within CMAP remains either inadequately explored or ambiguous. Furthermore, the database may not consistently offer drug perturbation profiles tailored to the lung or other pertinent cell types, a critical aspect for diseases like IPF. Incorporating a more detailed and expansive drug perturbation or drug target database could amplify UNAGI’s prowess in *in-silico* drug perturbation.

In summary, UNAGI is an AI-based computational tool that can be used to uncover distinct cellular trajectories during human disease progression, using distinct disease stages or severities or real-time course data, address regulatory and perturbation shifts that drive their phenotypes, and allow computational predictions of drugs that will reverse these shifts. We demonstrate its performance in a unique dataset of differentially affected tissues from patients with IPF, providing detailed observation, proteomic and experimental validations as well as relevance to another disease – COVID-19. We believe that the wide availability of UNAGI will enhance our understanding of complex diseases and accelerate the development of novel therapeutic strategies through the repositioning of known compounds, as well as the modeling of the effects of novel compounds.

## Methods

### Dataset description and preprocessing

The snRNA-seq IPF datasets were collected from a total of 19 individuals, comprising 10 healthy donors and 9 IPF patients. Recognizing that different regions of the lung may be at varying stages of disease progression^7^, we utilized cells isolated from these distinct regions within the IPF lung to model the temporal progression of IPF. Altogether, the dataset consists of 30 samples from control subjects and 24 samples from IPF patients. After sequencing, raw fastq files were trimmed with cutadapt^110^ (version 1.17) to remove read2 contamination of 5-prime template switch oligo and 3-prime polyadenylated tails; read pairs were discarded if read2 was trimmed below 30 bases. Trimmed reads were mapped to GRCh38 annotated with GENCODE^111^ (release 37) with the STARsolo^112^ implementation of STAR (v2.7.6a); the barcode whitelist file and barcode length parameters were based on the manufacturer’s (10X Genomics) guidelines for 3-prime v3.1 scRNAseq assays. Transcript count information was taken from STAR’s unfiltered ‘GeneFull’ output, all barcodes with at least 300 transcripts were imported into R (v 4.0.5) alongside statistics for each barcode’s percent of transcripts spliced, unspliced, or ambiguous from STAR’s ‘velocyto’ output. Cell barcode cleaning and cell type annotation were performed with tools from the R package Seurat (v4.1.1). To conduct an independent and manual cell type annotation, each sample of data was subjected to an iterative and recursive process of dimension reduction, graph embedding, and cluster analysis. After each iteration, the cell type labelling is refined, spurious nuclei are removed, and a subset of relatively similar cell types is isolated for the next iteration. This process was repeated recursively until all spurious nuclei were removed and new cell subpopulations could no longer be resolved. After each sample was cleaned and annotated, all samples were combined. Seurat’s reciprocal PCA integration method was used to adjust for batch effects at the cDNA library level. The gene expression results generated from integration were used for a final iteration of UMAP embedding and clustering. Final cell type assignments (the ‘Ground Truth’ column in Supplementary Fig 2) were determined by evaluating the true (not integrated) gene expression marker signatures of a cluster and confirming that the pattern was consistent across each sample. Following the preprocessing, we adopted the mesenchymal cell line which encompassed 231,544 cells to validate the UNAGI method. Please note that all subsequent analyses using the deep generative model solely utilize the normalized cell-by-gene matrix obtained from this preprocessing step. These analyses are augmented with manual cell type annotations performed independently via the Seurat pipeline, as detailed in this subsection. Crucially, all cell embeddings and clustering results presented in this manuscript were produced using our deep generative neural network framework, not the Seurat pipeline. The role of the Seurat framework was strictly confined to data preprocessing.

### Gaussian mixture density estimator

The Gaussian Mixture Model (GMM) clustering method^113^ leverages multivariate Gaussian components to characterize various stages of IPF samples. This approach aims to categorize samples into discrete stages of the disease, optimizing the probability or density representation of these stages. In this study, the GMM clustering approach is founded on the concept of surface density, serving as a measure of the extent of fibrosis. We assume that the surface density of all samples is independently and identically distributed, represented as *S*_*density*_ = {*s*_1_,*s*_2_, …. *s_n_*}, where *n* is the total number of samples. The GMM is fitted to the data to identify optimal components that maximize the log-likelihood: $P\left(S_{\text{density}}|\mu_{1,\ldots ,c},\sigma_{1,\ldots ,c}\right)=\sum_{i}^{c}\log 𝒩\left(S_{\text{density}}|\mu_{i},\sigma_{i}\right)$ Here, 𝒩 represents the Gaussian density function of the GMM for each sample, and *c* is the total number of Gaussian components, each characterized by a mean *μ*_*i*_ and standard deviation *σ*_*i*_. After identifying the optimal components, the samples *S* and their corresponding cells are softly classified into different stages, constructing the dataset *X* = *X*_1_, …, *X*_T_ where each $X\in R^{m_{T}\times n}$. Here, *T* refers to the number of IPF stages, and each stage has *m*_*T*_ cells with n genes.

### Graph Variational Autoencoder-Generative Adversarial Networks (Graph VAE-GAN)

Our UNAGI method introduces a Graph VAE-GAN model. To leverage cellular neighbors to diminish the effects of dropouts and noise^114^, we stacked a cell graph convolution (GCN) layer on top of VAE. A graph convolution layer is a specialized type of neural network that is can capture the topological structure of data, particularly by identifying features within local neighborhoods. GCN aggregates cell-cell relationships to construct a graph (*V*, *E*), where *V* denotes the vertices (cells) and *E* represents the edges (connections between cells). To establish this graph, the K-nearest neighbors (KNN) algorithm is employed to build the connectivity matrix *A* which defines the similarity between cells. The graph convolution is defined as *f*_*GCN*_(*X, A*) = *α*(*AXW*^*GCN*^), where *W*^*GCN*^ refers to the trainable weights of the GCN layer, and *α* is the activation function. Importantly, cells from different disease stages are not connected in the connectivity graph *A*, maintaining a stage-specific cell graph convolution.

UNAGI employs a VAE-based deep learning model^24^ to model the cellular dynamics behind complex disease progression and simulate the drug perturbations. The VAE’s encoder-decoder structure can model the probability distribution of high-dimensional data in a lower-dimensional space and generating new samples from this reduced-dimensional distribution. As a variational method, it facilitates the *in-silico* perturbation of cells by modulating their gene expressions. To refine the generative ability of VAE, we follow the previous method^116^ to use GAN to guide the generation of VAE with the min-max training strategy^115^. The encoder of the Graph VAE-GAN, *E*_*θ*_:*R*^*n*^ *→ R*^*l*^ consists of a GCN layer and several multi-layer perceptrons (MLPs). It can transform a cell ***x***_***i***_ ϵ *R*^*m*^ to its corresponding *l* -dimensional latent vector ***z***_***i***._ The GCN layer takes the normalized cell-by-gene count matrix *X* and connectivity matrix *A*, generating a graph representation *f*_*GCN*_(*X, A*) = *α*(*AXW*^*GCN*^) where *W*^*GCN*^ is weights of the GCN layer. Acknowledging that the latent distribution of single-cell data follows a multivariate normal distribution, two MLPs are employed to determine the means vectors *μ*_*z*_ = *f*_μ*_θ_*_(μ_*z*_|*f*_*GCN*_ (*X*, *A*)) and standard deviation vectors $\sigma_{z}=f_{\sigma_{\theta }}\left(\sigma_{z}|f_{GCN}(X,A)\right)$ of the latent representation. The latent representation for a cell is represented as $z\sim 𝒩\left(\mu_{z},\sigma_{z}^{2}\right)$ and the approximated posterior distribution is represented as *q*_*θ*_(*Z*|*X, A*).

The decoder *p_φ_*: *R*^*l*^ → *R*^*3n*^ takes *Z* as input to reconstruct the cell-by-gene count matrix. We employ the ZILN distribution to model the gene expression. The ZILN model is a composite distribution that integrates two distinct distributions: the first part is a Bernoulli distribution, Bernoulli(*ϱ*) which accounts for the dropout events commonly observed in single-cell sequencing. The second component of the ZILN model captures the actual gene expression levels following a log transformation, represented by log (*μ*, *σ*^2^). The likelihood function of a reconstructed cell ***x*** ϵ *X* can be written as:

(1) $$p_{\varphi }(x|z)=\prod_{j\in \text{genes}}\text{ZILN}\left(x_{j}|\varrho_{j},\mu_{j},\sigma_{j}^{2}\right)=\varrho_{j}\delta_{0}\left(x_{j}\right)+\left(1-\varrho_{j}\right)LN\left(x_{j}|\mu_{j},\sigma_{j}^{2}\right)$$

(2) $$LN\left(x_{j}|\mu_{j},\sigma_{j}^{2}\right)=\{\begin{array}{ll} \frac{1}{x_{j}\sigma_{j}\sqrt{2\pi }}e^{\frac{-{\left(\ln x_{j}-\mu_{j}\right)}^{2}}{2\sigma_{j}^{2}}}, & if\;x_{j}>0\\ 0, & if\;x_{j}=0 \end{array}$$

(3) $$\delta_{0}\left(x_{j}\right)=\{\begin{array}{l} 1,\;if\;x_{j}=0\\ 0,\;if\;x_{j}\neq 0 \end{array}$$

To reconstruct the cell-by-gene matrix *X*, the decoder *p*_*φ*_ will learn parameters of the ZILN distribution including the zero-inflation probability *ϱ* = *f*_*ϱϕ*_(*ϱ*|*Z*), scale of the log-normal distribution *σ* for each gene (a vector of for each gene (a vector of learnable parameters), and mean *μ* of the log-normal distribution, denoted as *μ* = *f*_*μϕ*_(*μ*|*Z*, *σ*) The prior distribution *p*(*Z*) is a multivariate standard normal distribution. Within our framework, we designate the entire Graph VAE model as the generator *G*. The loss function of the generator *L*_*G*_ can be formulated as:

(4) $$L_{G}=L(\theta ,\varphi ,X,A)=KL\left(q_{\theta }(Z|X,A)∥p(Z)\right)-E_{q_{\theta }(Z|X,A)}\left[\log p_{\varphi }(X|Z)\right]$$

The first term of *L*_*G*_ is the Kullback–Leibler divergences (KL), which quantifies the difference between the latent reconstruction *q*_*θ*_(*Z*|*X*, *A*) learned by the encoder and the predefined prior distribution *p*(*Z*). The second term is the expected log-likelihood of the input data given the reconstruction generated by the decoder, acting as a reconstruction loss. Together, *L*_*G*_ promotes the model’s generative performance with the probabilistic constraints of the latent space.

To further refine the generative capabilities of the Graph VAE, an adversarial discriminator is incorporated into the model’s architecture. This discriminator is a classifier based on MLPs to distinguish between original cells *X* and the reconstructed cells *G*(*X*, *A*) generated by the Graph VAE. A min-max adversarial training strategy is then applied, aimed at optimizing the loss function *L*_*GAN*._


(5) $$L_{GAN}=L(X,A)=\underset{G}{\min}\;\underset{D}{\max}\;E_{X}[\log(D(X))]+E_{X}[1-\log(D(G(X,A)))]$$

Here, *D* is the adversarial discriminator, G is the generator (Graph VAE). During the training phase, cells are labelled as real or fake (produced by the generator for the purpose of adversarial training). The discriminator, *D*, is optimized to effectively distinguish between real and fake cell labels, aiming to maximize the probability of correctly identifying real and generated cells. Simultaneously, the second term of *L*_*GAN*_ incentivizes the generation of cell reconstructions that are highly similar to the original data that *D* cannot distinguish them from real cells. The overall loss function of UNAGI denoted as *L*, is a composite of the Graph VAE loss and the GAN, written as *L* = *L*_*G*_ + *L*_*GAN*_. By integrating these components, UNAGI harnesses the strengths of various architectures, the GCN can leverage the cell-cell relationship information, the VAE can model the complex single-cell data, and the GAN can refine the quality of cell generation.

### Dynamics graph and underlying gene regulatory networks inference

UNAGI builds a dynamic graph to illustrate the progression of each cell population (cell type or subtypes) throughout disease progression. We apply Leiden clustering^117^ on the latent embeddings, generated by Graph VAE-GAN, to identify distinct cell populations at each disease stage. To measure distances between cell populations in adjacent stages, we use the KL divergence rather than Euclidean distance, which can be problematic in high-dimensional data contexts^118,119^. For each cell population (e.g., cell type), we approximate its distribution using a Monte Carlo Sampling strategy^120^ involving the sampling of each dimension of the latent embeddings a thousand times to form a multivariate normal distribution. The KL divergence is calculated to measure the distance between these populations’ multivariate normal distributions.

Additionally, we identify the top 100 differentially expressed genes (DEGs) in each cell population. We then calculate DEG distances among cell populations across stages. The DEG distance is defined as ${𝒯}_{d}\left(DEG_{c1},DEG_{c2}\right)*\sum_{j\in DEG_{c1}}\left|R_{j}^{c1}-R_{j}^{c2}\right|$, where the first term is the Jaccard Distance between *DEG*_*c_1_*_ and *DEG*_*c_2_*_, DEGs of two cell populations. The second term considers the ranking difference between two DEG lists. Here, $R_{j}^{c1}$ and $R_{j}^{c2}$ represent the ranking of gene *j* in *DEG*_*c_1_*_ and *DEG*_*c_2_*_, respectively. To render the KL divergence and the distances of differentially expressed genes (DEGs) comparable, we implemented min-max normalization for each metric across all potential connections within a specific cluster. After normalization, we represented the distances between each cluster pair as the sum of the normalized KL divergence and the normalized DEGs distances. We then compiled these normalized distances for all possible connections across various disease stages to create a background distance distribution. This distribution is essential for assessing the statistical significance of connections between clusters throughout the different stages of the disease. In scenarios where a cluster is connected to more than one cluster in an adjacent stage, the most statistically significant one will be used. These significant connections form tracks that trace from the control stage to the final stage of the disease, defining the disease progression. Consequently, the dynamic graph *G*_*dynamic*_produced includes these progression tracks, each representing comprehensive cellular state transition associated with a specific cell population during disease progression.

Moreover, we employ iDREM (Interactive Dynamic Regulatory Events Miner)^121^, a machine learning model based on an Input-Output Hidden Markov Model, to reconstruct the temporal gene regulatory network underlying each track (i.e., associated with each cell population) in the reconstructed cellular dynamics graph *G*_*dynamic*_. This gene regulatory network consists of co-expressed genes and gene regulators that regulate the temporal progression of the disease within each cell population. For each track in *G*_*dynamic*_, iDREM identifies the genes that undergo similar expression change patterns throughout the disease progression, which was termed as gene paths, some with increasing expression patterns while others with decreasing patterns. For each of the identified co-expressed gene paths, iDREM also provides its enriched GO terms and pathways. Beyond the identification of co-expressed gene paths, iDREM also captures the gene regulators that modulate those gene paths during disease progression. The dynamic genes and gene regulators identified through this process are considered dynamic marker candidates and hold potential as therapeutic targets for the disease.

### Iterative training strategy of UNAGI

The training strategy for UNAGI is structured as an iterative process, consisting of two primary phases that are cyclically repeated: (1) learning cell embeddings using the VAE-GAN framework, (2) constructing a cellular dynamics graph, and identifying substantial genes and gene regulators. Initially, with the cell embeddings learned with equal importance of all genes, we will employ the dynamics graph module to reconstruct the cellular dynamics and identify critical genes that influence disease progression, employing the iDREM algorithm. Based on this identification, UNAGI establishes and updates a gene-weights table for each cell. This table quantifies the importance of each gene and gene regulator, reflecting their roles in disease progression. As the training progresses through each iteration, dynamic marker genes and gene regulators that are deemed important in the reconstructed gene regulatory network are assigned increased weights. In contrast, genes not identified as critical or as gene regulators undergo a systematic decay in importance—specifically, a 30% decrement in weight with each iteration. This approach guarantees that genes consistently identified as critical in disease progression across various iterations receive progressively higher weights. Conversely, genes that are only occasionally deemed important will gradually lose prominence and be systematically deprioritized. Next, in the cell embedding learning of the subsequent iteration, the VAE model undergoes fine-tuning with a modified loss function that accentuates the high-weight genes. This enhancement is accomplished by integrating the gene weights in all cells into the reconstruction loss function, thereby shifting the model’s focus from generic genes to those disease-associated genes identified through gene regulatory network inference. During each iteration, after the cell embeddings are updated, the cellular dynamics module steps in to rebuild the cellular dynamics graph and the associated gene regulatory networks. This stage plays a crucial role in refining and updating the disease-associated genes. These enhancements feed back into and improve the cell embedding learning in the next iteration. On the other hand, the revised cell embeddings generate an updated cellular dynamics graph and its gene regulatory network, offering a deeper understanding of disease progression and potentially advancing the identification of disease-specific genes, which will in return improve the cell embedding learning in the next iteration.

Upon model convergence, the highest-weighted genes are associated with the disease and thus indicating that UNAGI model can indeed ‘comprehend’ the disease and recognize important disease-relevant genes during the iterative training. For instance, enrichment analysis shows that the top 100 weighted genes in fibrotic fibroblast cells are closely associated with IPF (Supplementary Fig. 12). At each training iteration *t,* the gene weights are transformed into a ranking matrix, *R*^*t*^. The objective functions of UNAGI during its iterative training can be then refined as follows to integrate the distilled disease knowledge in the gene-weights table for each cell:

(6) $$L_{G}^{t}=L\left(\theta^{t},\varphi^{t},X,A\right)=KL\left(q_{\theta^{t}}(Z|X,A)∥\text{p}(Z)\right)-E_{q_{\theta }t}(Z|X,A)\left[\log p_{\varphi^{t}}(X|Z)\cdot \frac{1}{{\left(R^{t}\right)}^{\tau }}\right]$$

(7) $$L_{GAN}^{t}=L(X,A)=\underset{G^{t}}{\min}\;\underset{D^{t}}{\max}\;E_{X}\left[\log\left(D^{t}(X)\right)\right]+E_{X}\left[1-\log\left(D^{t}\left(G^{t}(\text{X},\text{A})\right)\right)\right]$$

(8) $$L^{t}=L_{G}^{t}+L_{\text{GEN}}^{t},t\in (0,1,\ldots ,T)$$

Here, G^t^ represents the generator at the *t*-th iteration, and *D*^*t*^ is the discriminator at the same iteration. $L_{G}^{t}$ denotes the loss of generator, and $L_{GAN}^{t}$ denotes the loss of GAN at the *t*-th iteration and *τ* is a hyperparameter which is responsible for controlling the influence of gene weights on the reconstruction loss (empirically set *τ* as 0.5). Through this iterative training, UNAGI progressively hones its ability to generate disease-specific cell embeddings. This approach allows for the identification of disease-specific markers and supports disease-specific *in-silico* perturbations.

### Dynamic markers discovery

To characterize the temporal progression of the disease for each cell population, we identify dynamic markers. They are genes that change considerably throughout the disease’s progression. For each track in the cellular dynamics graph, iDREM identifies the gene paths with co-expression patterns during disease progression as discussed above. Next, we computed the sum of fold changes for each gene in each of the gene paths across all disease stages associated with each cell dynamic track (i.e., a cell type or subtype). Genes in those identified gene paths were considered as candidate dynamic biomarkers for further statistical examinations. To calculate the statistical significance of the candidate markers, we randomly shuffle cells within each stage of the track to generate the background simulation tracks. We then calculate the accumulated foldchanges among all neighboring stages of these candidate markers across all simulation tracks. This simulation process was repeated *N* times (*N* >1000) to establish a random background foldchange distribution. We then evaluated the P-values for each candidate marker based on its accumulated sum fold change against this background distribution. To ensure a high level of confidence in our selection of dynamic markers, we imposed a more stringent FDR cut-off (FDR < 0.01) than the default (FDR <0.05). These selected dynamic markers are pivotal in delineating the progression tracks and provide a nuanced understanding of the longitudinal evolution of the disease within each distinct cell population.

### Hierarchical static markers discovery

The hierarchical static marker discovery approach supports the identification of intra-stage static markers through hierarchical clustering. UNAGI conducts hierarchical clustering based on the embeddings of cell populations at each disease stage, thereby generating dendrograms to depict the relationships among these populations. Initially, we identify distinct cell populations using their latent embeddings *Z*. Hierarchical clustering is then applied to these embeddings to construct a dendrogram for the cell populations within the same disease stage. This dendrogram serves as a tool to explore the hierarchical structure of cell populations. In this dendrogram, when focusing on a particular cluster, we analyze it at various levels to identify hierarchical static markers. At lower levels of the dendrogram, the selected cluster compares with a broader range of sibling clusters. Here, the hierarchical static markers identified tend to represent general features of the cell population, as the siblings encompass a wider scope. For instance, at level 0, the selected cluster is compared against all other clusters within that stage. Conversely, at higher dendrogram levels, the siblings are more closely related to the selected cluster. This closeness allows for the identification of markers that highlight the subtle heterogeneities among cell subpopulations within the same cell type. By examining these nuanced differences, we can gain a deeper understanding of the cell subpopulation’s characteristics.

### *In-silico* perturbation strategies

*In-silico* perturbation can be executed through two strategies: (1) Direct gene expression regulation. This approach involves the direct up-regulation or down-regulation of specific genes of interest. For a cluster of cells, we define an expression regulation vector $\Delta =\left[\Delta_{g1},\Delta_{g2},\ldots ,\Delta_{gn}\right]$, where each Δ_*gn*_ represents the expression change of gene 𝑔𝑛 (e.g. Δ_*g*1_= 0.5 would indicate an increase in the expression of gene *g*1 by 0.5). The gene expression for a perturbed cell population *X′*_*c*_ can be defined as:

(9) $${X^{\prime }}_{C}=\text{max}\left(X_{C}+1_{M_{c}}\Delta ,0\right)$$

Here, *X*_*c*_ represents the original cell-by-gene matrix of a cell population *c*, and *M*_*c*_ represents the number of cells within the cell population. (2) Gene Interaction (GI) Network-based regulation. In this strategy, we regulate the genes of interest and their interacting partners based on the gene interaction network curated from public domains. From the HIPPIE database^122^ and STRINGDB^123^, we obtain the strength of gene interactions *γ* of different gene pairs. For a certain cell population *c*, we transformed the cell-by-gene matrix *X*_*c*_ into a gene-by-cell matrix *Y*_*c*_ and employed PCA to generate low-dimensional embeddings *P*_*gene*_ for each gene across the cell population. The influence factor *I*(*Q*, *R*) ϵ (−1,1) quantifies the extent to which the perturbation of a given gene *Q* impacts on another gene *R*. *I*(*Q*, *R*) is defined as:

(10) $$I(Q,R)=\left\{\begin{array}{c} 0,\;\text{ if Q and R are not connected}\\ sgn\left(cor\left(y_{Q},y_{R}\right)\right)\exp\left(-w\frac{∥P_{Q}-P_{R}{∥}_{2}}{\prod_{k\in (Q,R)}\gamma_{k}}\right),\;\text{otherwise} \end{array}\right.$$

(11) $$\text{sgn}(x)=\{\begin{array}{c} 1,x>0\\ 0,x=0\\ -1,x<0 \end{array}$$

Here, ***y***_*Q*_ and ***y***_*R*_ are gene expression vectors of genes *Q* and *R*, respectively, in the *Y*_*c*_. The term (*Q*, *R*) denotes a sequence of hops from *Q* to *R* in the GI network, *γ*_*k*_ denotes the strength of gene interactions of a hop in (*Q*, *R*), *w* is the steepness weight (*w* > 0 and empirically set to 0.2 by default) to control the influence factor, *cor*(***y***_*Q*_, ***y***_*R*_) quantifies the correlation between two genes and *sgn*(*x*) indicates the direction of their interactions. The gene of interest will tend to impose higher impacts on genes that it directly interacts with. Conversely, genes that are further away in the GI network are less influenced. When regulating a specific gene *η* by changing a certain magnitude Δ_*η*_ (e.g., Δ_*η*_ = −0.5 can decrease the expression of gene *η* by 0.5). The expression regulation vector for this scenario is formulated as $\Delta =\left[\Delta_{\eta }I\left(\eta ,g_{1}\right),\Delta_{\eta }I\left(\eta ,g_{2}\right),\ldots ,\Delta_{\eta }I\left(\eta ,g_{n}\right)\right]$. If multiple genes *G*_*P*_ are perturbed with individual magnitudes, the expression regulation vector is:

(12) $$\Delta =\left[\sum_{i\in G_{P}}\Delta_{i}I\left(i,g_{1}\right),\sum_{i\in G_{P}}\Delta_{i}I\left(i,g_{2}\right),\ldots ,\sum_{i\in G_{P}}\Delta_{i}I\left(i,g_{n}\right)\right]$$

The gene expression for a perturbed cell population *X*′_*c*_ is then calculated as defined in equation (9).

### *In-silico* perturbation scoring

We perform perturbations on every stage of individual tracks using the perturbed cell-by-gene expression matrix *X*’. This matrix *X*’ is fed into the encoder of the Graph VAE-GAN, yielding the perturbed latent cell representation $Z^{\prime }=E_{\theta }\left(X^{\prime },A\right)$. The efficacy of these perturbations is assessed by examining the changes in the distances between cell populations within the latent cell embedding space. Specifically, the distance between two cell populations in the latent space Z can be quantified as $\delta_{{\text{i}}^{\prime },\text{j}}={\left\|Z_{i}^{\prime }-Z_{j}\right\|}_{2}$, where *i* is the perturbed cell population and *j* is another cell population within the same track. The perturbation score of a track *S*_*track*_ ϵ [−1, 1] at a perturbed stage *i* is defined as:

(13) $$S_{\text{track}}(i)=\frac{1}{T}\sum_{j=0,j\neq i}^{T}\left(1-\frac{2}{1+\text{exp}\left(w\left(\delta_{i\prime ,j}-\delta_{i,j}\right)\text{sgn}(j-i)\right)}\right)$$

Here, *T* represents the total number of stages, *i* is the perturbed stage, *w* is a hyper-parameter to control the scaling (*w* is set as 100 in our case), *δ*_*i,j*_ is the distance between stage *j* and *i* (unperturbed) and $\delta_{i\prime ,j}$ is the distance between stage *j* and *i* (perturbed). The function *sgn*(*x*) (as defined in equation (11)) is a perturbation indicator function to ensure the perturbed cell population that comes closer to the control stage will always have a positive and higher score while moving away leads to a negative and lower score. In addition to track-level perturbation scoring, an overall score *S* assesses perturbation effects across all tracks. This overall score is normalized based on the proportion of cells in each perturbed track within the dataset. It also incorporates the gene-regulating directions of compounds, as indicated in the relevant database, including their reversed directions. The overall score *S* for all stages is defined as follows,

(14) $$S=\sum_{h\in \text{tracks}}\frac{N_{h}}{N}\sum_{i\in \text{stages}}\frac{\left|S_{h}^{𝒜}(i)-S_{h}^{ℬ}(i)\right|}{2}$$

where 𝒜 represents the perturbation direction that aligns with the reported direction of the drug target expression change, while *ℬ* denotes the opposite drug target expression change direction as reported in the CMAP database. The overall score 𝒮 is calculated by considering in-silico perturbations in both directions, enhancing robustness. This approach is based on the premise that perturbing the targets of an effective drug in opposite directions should lead to a higher $S_{h}^{𝒜}(i)$ and lower $S_{h}^{ℬ}(i)$, resulting in an increased score S. *N* here is the total number of cells and *N*_*h*_ is the number of cells in the perturbed track.

### Therapeutic pathways discovery

We use pathway data from REACTOME^124^, MatrisomeDB^125^, and KEGG^126^ databases, providing lists of genes associated with various biological pathways. The set of genes present in individual single-cell transcriptomics datasets might vary, especially after data preprocessing. Therefore, we used expressed genes after preprocessing, and are listed as pathways’ targets for *in-silico* pathway perturbations. We applied the scoring and ranking strategies as discussed in the ‘In-silico perturbation strategies’ and ‘In-silico perturbation scoring’ sections above to identify potential therapeutic pathways. Pathways that do not share any genes with our processed single-cell data are excluded from this analysis, as they cannot be effectively evaluated. To assess the significance of our *in-silico* pathways perturbations, we establish a random background dataset by randomly sampling 𝑛 genes 1000 times where *n* is set to the median number of genes across all pathways. The perturbation strength Δ used for random background perturbations was matched to that employed for the actual pathway in-silico perturbations. We executed *in-silico* perturbations using the random dataset described above to generate a random background therapeutic score distribution. By contrasting the perturbation scores with this background distribution, we could ascertain the statistical significance of the *in-silico* pathway perturbations. This approach aids in identifying potential therapeutic pathways with a false discovery rate (FDR) of less than 0.05.

### Candidate drugs and compounds discovery

We use the compound and their target genes from the Connectivity Map (CMAP) database^26,27^ which contains 34, 396 compound or drug profiles. Similar to the pathway perturbation, we used expressed genes after preprocessing, and are listed as drugs’ targets for *in-silico* drug perturbations. We applied the scoring and ranking strategies as discussed in the ‘In-silico perturbation strategies’ and ‘In-silico perturbation scoring’ sections above to identify potential drug candidates. The method for calculating the statistical significance of in-silico drug perturbations was akin to that used for therapeutic pathway perturbations, as mentioned previously. The primary distinction lies in the number of genes selected for creating the random background dataset and the perturbation strength Δ, which was aligned with that of the actual drug perturbations.

### Clustering parameters optimization

To maintain consistency in cluster numbers and sizes, as well as the distances between cell neighbors, across various stages, we introduce a Clustering Parameter Optimization (CPO) method. Connectivity graph-based community detection clustering methods, like Leiden clustering^117^, can automatically identify the number of clusters. However, the improper number of neighbors in the neighborhood graph or the improper resolution setting can lead to over-clustering or under-clustering, introducing complications in the analysis process. The consistency in the number and size of clusters is important for tracing the lineage of cell populations through various stages of development or disease progression. The proposed CPO method encompasses two primary steps. (1) Searching for the optimal number of neighbors to construct graphs with consistent cell-neighbor distances across different stages. We start by selecting an anchor stage, which is the stage with a cell count closest to the median count of all stages, denoted as *N*_*anchor*_. We then calculate the average distance between cells and their neighbors in this anchor stage, establishing the anchor neighbor distance. The goal for other stages is to find a number of neighbors that yields a neighbor distance similar to that of the anchor stage. (2) The second step involves determining the optimal clustering resolution. We aim to find a set of resolutions within the predefined range [*R*_*min*_ = 0.8, *R*_*max*_=1.5] for different stages that result in a similar median number of cells per cluster across these stages. For different application scenarios, users have the option to select a resolution range larger than the default setting. This flexibility enables adaptation to various analytical needs and preferences. By employing the CPO method, we ensure that the neighborhood graphs for different stages maintain similar cell-neighbor distances. Additionally, this approach ensures a consistent number and size of clusters across different stages, thereby enhancing the coherence and robustness of our analytical framework.

### Benchmarking

To evaluate UNAGI’s performance in learning latent embeddings from single-cell data, it is benchmarked against scVI^15^ and SCANPY^12^. SCANPY applies Principal Component Analysis (PCA) for dimensionality reduction, whereas scVI uses a VAE model to capture the latent structures of single-cell data. We employ non-label metrics including the Silhouette score^127^, which assesses cluster cohesion and separation, Davies-Bouldin index^128^, which gauges average similarity ratios between clusters, and Label score^89^, which evaluates the cell type consistency in the cell neighborhoods. For labeled metrics, the independent manual cell-type annotation served as a reference for calculating ARI^87^ and NMI^88^. In the benchmarking and ablation experiments, we compare UANGI with scVI, SCANPY, the baseline VAE model (BL) using ZILN distribution, and the baseline model with GCN (BL_GCN) and GAN (BL_GAN), respectively. To ensure a fair comparison, we run each method for 15 rounds, each with different random seeds, and utilize Leiden clustering to generate the clustering results. Furthermore, the effectiveness of the ZILN distribution in modeling rigorously normalized single-cell data was evaluated by comparing scVI-ZILN with standard scVI. To assess UNAGI’s iterative training strategy, it was executed five times with different seeds, and the results from the initial five iterations were benchmarked.

### *In-silico* drug discovery simulation

We developed a simulation dataset to assess the ability of UNAGI to identify potential therapeutic targets. We selected drugs and compounds that are least likely (set FDR>0.95 as the default cut-off) to be effective in disease medication. For the generation of positive simulation data, we manipulated the target gene expression levels across various disease stages. Specifically, for compounds known to down-regulate their targets, we set their target expression levels as a progressive series [*B*, 2*B*, . . , *T* * *B*], where *B* > 0 at each disease stage (in total, we have *T* disease stages). Conversely, for compounds that up-regulate their targets, the expression levels were set in a decremental series [*T* * *B*, (*T* − 1) * *B*, …, *B*]. In the context of negative data, we maintained consistent gene expression levels without any alteration. In the *in-silico* drug perturbation, the drug will increase the expression to ϵ times or decrease to its 1/*ϵ*. We conducted experiments in four separate simulation rounds, each utilizing different *B* values (0.5, 1.0, 1.5, 2.0). Within each round, we explored the effects of four distinct *ϵ* values (1.5,2.5,3.5,4.5). We established a random background score distribution by randomly sampling *n* genes 1000 times, where *n* is set to the median number of genes across all perturbed drugs. The drug candidates with a significant FDR cut-off (FDR<0.01) than the default 0.05 were considered as successfully predicted. The model’s performance in drug discovery was assessed using the Area Under the Receiver Operating Characteristic Curve (AUROC) and the Area Under the Precision-Recall Curve (AUPRC) metrics, as implemented in scikit-learn129.

### Sanity perturbation approach

To evaluate the effectiveness of our proposed *in-silico* perturbation strategy, we also employed a sanity perturbation approach. This involved randomly selecting a specific track from the temporal dynamics graph and calculating the average gene expression at each stage along this track to determine the centroid for each stage. For all stages other than the control, we adjusted the gene expression of all cells within each stage to match that of the preceding stage. This was achieved by subtracting the centroid differences between these stages on each of the cells from the stage. Subsequently, the perturbed cells were input into the UNAGI model, and following the ‘In-silico perturbation strategies’ and ‘In-silico perturbation scoring’ sections above to obtain the perturbed cell embeddings. We then calculated the perturbation scores, which served as a metric to evaluate the effectiveness of our perturbation strategy. Sanity perturbations should result in positive and far larger therapeutic scores compared to random perturbations.

### Verify UNAGI biomarkers by proteomics data

Proteins were extracted from pulmonary tissues using the MPLEx protocol as previously described^130–133^. Thirty tissue blocks from IPF donors and 10 from control donors were employed. Briefly, the tissue samples were then homogenized using a Qiagen TissueLyser II with a 2 × 24 adapter (chilled to −20°C) following vendor’s instruction^133^. The aqueous polar metabolites and proteins were extracted from each homogenate using the MPLEx protocol. Keeping each sample on ice, a volume of chilled chloroform and water were added to a final ratio of 3:8:4 water-chloroform-methanol, mixing gently after each addition. The samples were chilled on ice for 5 min before mixing well and separating the layers by centrifugation (10,000 g, 10 min, 4°C). Proteins were isolated and concentrated to dryness in a vacuum concentrator and stored at −70°C until ready for further processing. For each sample proteins were denatured, alkylated, digested with trypsin, and desalted on a C18 solid-phase extraction (SPE) cartridge using previously detailed methods^132^. For the analyses, 5 μL of resulting peptides at the concentration of 0.1 μg/μL were analyzed by reverse phase liquid chromatography coupled with an Orbitrap Lumos instrument (Thermo Scientific) in data-dependent mode (DDA). Briefly, samples were loaded on an SPE column via a 5 μL sample loop separated by the C18 column using a 120 min gradient. The effluents from the LC column were ionized by electrospray ionization and introduced into the mass spectrometer via a heated capillary maintained at 250°C for ion desolvation. The resulting ions were mass analyzed by the Orbitrap at a resolution of 60,000 covering the mass range from 300 to 1,800 Da. The top 12 most intense ions were then targeted for fragmentation per cycle time. Tandem mass MS2, ions were isolated by quadrupole mass filter in monoisotopic peak selection mode using an isolation window of 0.7 Da, maximum injection time of 50 ms with AGC setting at 1E5 ions and fragmented by high-energy collision dissociation (HCD) with nitrogen at 32% normalized collision energy. Fragment ions were mass analyzed by the Orbitrap at a resolution of 7,500, and spectra were recorded in the centroid mode. Ions once selected for MS2 were dynamically excluded for the next 45s. The instrument raw files are publicly available on MassIVE (Server: massive.ucsd.edu, User: MSV000093129, Password: Lung5172). The raw files were analyzed using MaxQuant v1.6.0.16 LFQ quantification. Downstream data processing and statistics were performed using RomicsProcessor^134^. The resulting LFQ intensities were log2 transformed and median-centered. ANOVA and Student’s T-test were performed, and FDR p-value corrections were applied. The data processing code is available on GitHub (https://github.com/GeremyClair/IPF_DDA_proteomics/).

After preprocessing, we adopted a more stringent FDR cut-off (FDR<0.01) than the default (FDR<0.05) to identify highly confident dynamic proteins. To verify the temporal dynamic markers determined for each progression track, we applied hypergeometric testing. This test assessed the overlapping ratio between dynamic proteins and dynamic markers. The overlapping between these two marker lists associated with a track is considered statistically significant if the FDR from the hypergeometric test is less than 0.05. We then use heatmaps to visualize the LFQ intensities and gene expression from proteomics data and snRNA-seq data, respectively.

### Precision-cut lung slice (PCLS) experiments

Fresh lung tissue of explanted donor lungs was used for human PCLS according to previously published protocols^37,90,135^. Donor lung samples were sourced from 6 males and 4 females and were obtained from the Center for Organ Recovery and Education (CORE) at the University of Pittsburgh. Donor lung samples originated from lungs deemed unsuitable for organ transplantation. For the fibrosis induction in hPCLS, PCLS were treated for 5 days with a control cocktail (CC) including all vehicles or a pro-fibrotic cocktail (FC) consisting of TGF-β (5 ng/ml, Bio-Techne), PDGF-AB (10 ng/ml, Thermo Fisher), TNF-α (10 ng/ml, Bio-Techne), and LPA (5 *μ*M, Cayman chemical) as described before^90,136^. For drug treatments, PCLS were treated with FC allowing for the induction of fibrosis, and drug treatment started at day 3 until day 5. At the end of the experiment, PCLS were snap-frozen individually in liquid nitrogen for single nuclei analysis, as described above. The study was approved by the University of Pittsburgh (IRB PRO14010265). Written informed consent was obtained for all study participants. Nuclei were extracted using the Nuclei Isolation kit (CG000505, 10X Genomics,). 20’ 000 nuclei were loaded on a Chip G with Chromium Single Cell 3′ v3.1 gel beads and reagents (3′ GEX v3.1, 10x Genomics). Final libraries were analyzed on an Agilent Bioanalyzer High Sensitivity DNA chip for qualitative control purposes. cDNA libraries were sequenced on a HiSeq 4000 Illumina platform aiming for 150 million reads per library and a sequencing configuration of 26 base pair (bp) on read1 and 98 bp on read2.

Basecalls were converted to reads with the software Cell Ranger’s^137^ (v4.0.0) implementation mkfastq. Multiple fastq files from the same library and strand were catenated to single files. Read2 files were subject to two passes of contaminant trimming with Cutadapt^110^ (4.1) for the template switch oligo sequence anchored on the 5′ end and for poly(A) sequences on the 3′ end. Following trimming, read pairs were removed if the read2 was trimmed below 30 bp.

Paired reads were filtered if either the cell barcode or unique molecular identifier (UMI) sequence had more than 1 bp with a phred of <20. Reads were aligned with STAR^112^ (v2.7.9a) to the human genome reference GRCh38^111^ release 99. After preprocessing, analysis of the ex vivo human PCLS snRNA-seq data was conducted using the Seurat^11^ package (version 1.8.2). Cells with less than 750 transcripts profiled were then removed.

To minimize the possible effect of potential batch correction methods, we first processed and annotated each library separately, before integrating them together and annotating them jointly. To integrate the multiple snRNA-seq datasets, we employed Robust Principal Component Analysis (RPCA)^138^. Based on the cellular diversity, we chose to use PCLS treated with DMSO as the reference for the integration. Following the RPCA decomposition, we utilized the low-rank component as the integrated representation of the snRNA-seq datasets. This component captured shared biological signals across conditions while mitigating dataset-specific variations. Subsequent analyses, such as clustering and differential expression analysis, were performed on the non-integrated but normalized gene expression values. To validate the effectiveness of the integrated representation, we performed various analyses, including cell-type clustering, and identification of marker genes. We also compared the results of these analyses to those obtained from individual datasets to evaluate the improvement gained through the integration process. Marker genes were computed using a Wilcoxon rank-sum test, and genes were considered marker genes if the FDR-corrected p-value was below 0.05 and the log2 fold change was above 0.5.

We then applied the Graph VAE-GAN to learn the latent embeddings of the PCLS data. To quantify the effects after treating the fibrosis cells with the drugs, we calculate the pairwise Euclidean distance from control cells to real treatment cells and fibrosis cells in the reduced latent space. We used the difference between the centroid of fibrosis cells and centroids of real treatments as the perturbation strength vector Δ. We conducted in-silico drug perturbations on fibrosis cells using a consistent perturbation strength Δ. The efficacy of these in-silico perturbations was evaluated through UMAP visualizations and by measuring the pairwise Euclidean distances between cell embeddings in latent space. Our primary objective was to ascertain if in-silico drug perturbations could replicate the cell embeddings in latent space as observed with actual drug treatments, thereby validating the accuracy of UNAGI-driven in-silico drug perturbations. Additionally, to compare the similarity of the differential genes associated with the in-silico drug perturbations (in-silico drug perturbation vs. fibrosis) and those of real drug treatment (drug vs. fibrosis), we employed Ranked-Ranked Hypergeometric Overlap (RRHO) plots. Moreover, box plots and the *R*^2^ score were used as analytical tools to quantify gene expression similarities between cells under actual drug treatments and cells produced from our in-silico perturbations for both Nintedanib and Nifedipine.

## Data Availability

IPF snRNA-seq and PCLS data will be made publicly accessible upon the publication of this work. The COVID-19 dataset (COVID-19 PBMC Ncl-Cambridge-UCL) is currently available from the COVID-19 Cell Atlas at https://covid19cellatlas.org/. The proteomics data are publicly available on MassIVE (Server:massive.ucsd.edu, User: MSV000093129, Password: Lung5172).
